# Effective psychological therapies to improve lifestyle behaviors in (pre)pregnant women: A systematic review

**DOI:** 10.1016/j.pmedr.2021.101631

**Published:** 2021-11-09

**Authors:** M. van der Windt, S.K.M. van Zundert, S. Schoenmakers, P.W. Jansen, L. van Rossem, R.P.M. Steegers-Theunissen

**Affiliations:** a Department of Obstetrics and Gynecology, Erasmus MC, University Medical Center, Rotterdam, Netherlands; b Department of Child and Adolescent Psychiatry/Psychology, Erasmus MC, University Medical Center, Rotterdam, Netherlands; c Department of Psychology, Education and Child Studies, Erasmus University Rotterdam, Netherlands

**Keywords:** Pregnancy, Lifestyle behavior, Psychological therapy, Nutrition, Substance use

## Abstract

Poor lifestyle behaviors impact (pre)pregnant women by affecting pregnancy outcomes and offspring health. This systematic review provides an overview of psychological therapies to support lifestyle behavior changes among (pre)pregnant women.

Scientific databases were searched from their inception to 20 December 2020 for studies investigating the effects of psychological therapies on improvements in lifestyle behaviors.

Studies were eligible if they included (pre)pregnant women, examined the effects of a psychological therapy on at least one lifestyle behavior and used a control group receiving usual pregnancy care or a non-psychological intervention. Lifestyle behaviors of interest were dietary intake, physical activity, smoking, alcohol consumption, drug use, body weight loss and body weight gain during pregnancy. Pregnancy complications were included as outcome measures.

Motivational interviewing (MI) (n = 21), cognitive behavioral therapy (CBT) (n = 8), incentive-based contingency management (IBCM) (n = 9), mindfulness (n = 1) and hypnosis (n = 1) were investigated as lifestyle behavior interventions. The findings revealed that MI was effective in reducing (self-reported) smoking and alcohol consumption and restricting gestational weight gain (GWG). CBT was only studied as an intervention to restrict GWG and the results predominantly confirmed its effectiveness. IBCM showed the strongest effect on reducing smoking and substance use. The studies using hypnosis or mindfulness to reduce smoking or restrict GWG, respectively, showed no associations.

The use of psychological therapies to improve lifestyle behaviors among (pre)pregnant women is new and the scientific proof is promising. Before wide implementation is legitimated, more evidence is needed on the consequences of lifestyle change for pregnancy outcomes.

## 1 Introduction

Having a healthy lifestyle is of great importance for women before and during pregnancy. Remarkably, only 7–15% of women of reproductive age adheres to healthy lifestyle behaviors (van der Windt et al., 2020, Zhao et al., 2012). Poor lifestyle behaviors during the periconception period impact (pre)pregnant women by affecting reproductive and pregnancy outcomes and offspring health. Additionally, exposing the developing fetus to an unfavorable environment in utero can cause transgenerational health effects (Gluckman et al., 2008). Thus, for (pre)pregnant women in particular, it is crucial to have a healthy lifestyle, since it affects both the individual’s well-being as the health of future generations.

A healthy lifestyle comprises a combination of behaviors that contribute to lower morbidity and mortality and a better quality of life (Li et al., 2020). In general, following a healthy diet, drinking limited amounts or even quit consumption of alcohol, not smoking, no usage of drugs, and regular exercises are essential components of a healthy lifestyle (Li et al., 2018, Loef and Walach, 2012). Additionally, having a normal body mass index (BMI) (18.5–24.9 kg/m^2^) is considered as an essential component of a healthy lifestyle as well as a result of an adequate balance between nutritional intake and physical exercise (Peeters et al., 2003). For (pre)pregnant women as well, these lifestyle behaviors are essential for positive pregnancy outcomes and for the prevention of pregnancy complications (Hill et al., 2020).

Several proven effective lifestyle interventions have been developed to support the improvement of lifestyle behaviors in (pre)pregnant women (Oteng-Ntim et al., 2012, Van Dijk et al., 2016). However, adopting healthy lifestyle behaviors is challenging and interventions often do not lead to satisfactory results and sustainable change. Most lifestyle-targeted interventions focus on increasing external motivation by raising awareness and providing education, but lack elements that increase intrinsic motivation and support lifestyle change on the long term (Brandt et al., 2018, Lachman et al., 2018). In recent years, psychological therapies, as cognitive behavioral therapy, mindfulness, and contingency management, have increasingly been used in lifestyle interventions to improve lifestyle behaviors (Brandon, 2014, Haug et al., 2014). These psychological approaches intend to increase intrinsic motivation and to teach the participants skills including impulse control techniques, cognitive restructuring and problem-solving strategies to enhance change in lifestyle behaviors. Recently, a variety of psychological therapies have been investigated as lifestyle interventions for (pre)pregnant women (Blau and Hormes, 2020). However, no study performed a systematic review of the available literature on this subject. The current systematic review provides a unique overview that can be used for maternal preconception health improvements in daily clinical practice. We aim to explore which psychological therapies have been proven as effective interventions towards improving lifestyle behaviors and pregnancy outcomes among (pre)pregnant women.

## 2 Methods

Our systematic review was performed in accordance with the Preferred Reporting Items for Systematic Reviews and Meta-analyses (PRISMA) guidelines (Liberati et al., 2009, Page et al., 2021). A protocol of our systematic review was registered in PROSPERO International prospective register of systematic reviews (registration number: CRD42020201172).

### 2.1 Search strategy and information source

In consultation with an experienced information specialist, we developed Boolean search strategies including terms as pregnancy, preconception, smoking, alcohol, drugs, nutrition, physical activity, (cognitive) behavioral therapy, incentives, motivational interviewing, motivational enhancement therapy, mindfulness, hypnotherapy, maternal complications, mode of delivery, neonatal outcome, fetal malformations, gestational age at delivery, and birth weight (Appendix). We searched for clinical trials in the following databases: Embase, Medline (Ovid), Web of Science, PsycINFO, Cochrane Central Register of Controlled Trials, Google Scholar (top 200), all from their inception to 20 December 2020. Finally, we searched reference lists from included studies and systematic reviews to include relevant articles. We did not search gray literature, due to a lack of reproducibility and quality concerns (Adams et al., 2016).

### 2.2 Eligibility criteria and study selection

Studies were included if they met the following criteria: 1) included women contemplating pregnancy or already pregnant 2) examined the effects of a psychological therapy on at least one lifestyle behavior, 3) used a control group receiving usual pregnancy care or a non-psychological intervention. Studies without a clear definition of the tested psychological therapy were excluded. Lifestyle behaviors included dietary intake, physical activity, smoking, alcohol consumption and drug use, but also stress, sleep, and psychological state of mind, are considered as lifestyle behaviours (Abe and Abe, 2019). However, we decided to focus on factors not directly related to or representing mental health, since psychological therapies are widely investigated and proven effective for improving those factors. In general, BMI, and gestational weight gain (GWG) in particular, do not directly reflect dietary intake. However, they are considered as a composite outcome of lifestyle behaviors (Itani et al., 2020, Sun et al., 2020). Therefore, BMI and GWG are included as lifestyle behaviors in our systematic review.

Letters to the editor, conference abstracts, editorials, opinions, case reports and systematic reviews were not eligible. We did not apply a language limitation to our search strategy. Two independent reviewers examined each article for inclusion. If the two reviewers disagreed on whether to include an article, a third reviewer was consulted to resolve any disagreements.

### 2.3 Data extraction and assessment of risk of bias

The two reviewers filled out a data extraction form and used the ErasmusAGE quality assessment tool for assessing risk of bias of the individual studies. This tool is composed of 5 items based on previously published scoring systems (Thomas et al., 2004). Five study characteristics can be allocated either 0, 1, or 2 points giving a total score between 0 and 10, with a score of 10 representing a study of the highest quality.

### 2.4 Data synthesis

Results are presented in a narrative synthesis for each type of psychological therapy and displayed in several tables. It was not possible to perform a *meta*-analysis due to the large heterogeneity of content and intensity of the psychological therapy interventions.

Relative risks (RRs) were collected from all included studies and presented in a forest plot. RRs were calculated when not incorporated in the results of the included study, if required data were available. When studies compared three groups, the most intensive intervention, in frequency (number of counseling sessions) and intensity (length of counseling sessions), was compared with the least intensive intervention or the control group.

## 3 Results

### 3.1 Study selection

**Fig. 1:**
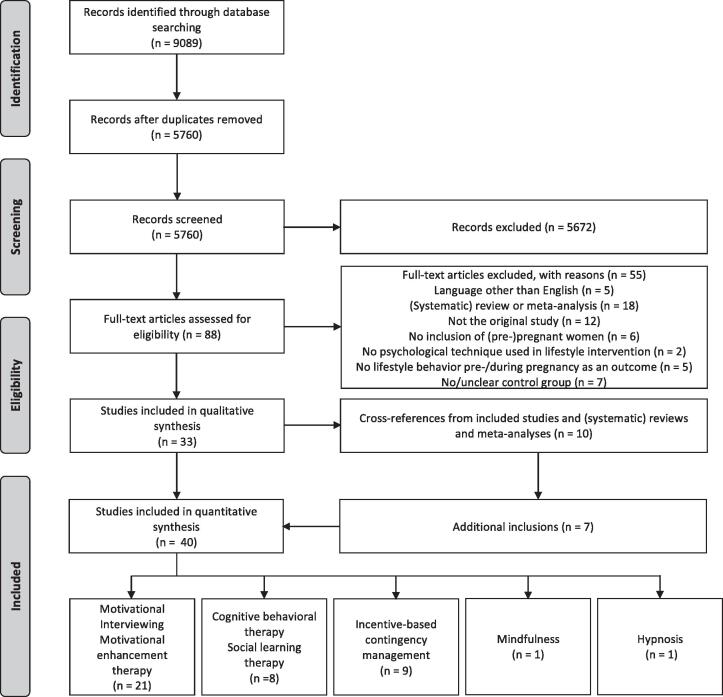
Flowchart of studies included in the current systematic review.

The study selection process is depicted in the flowchart (Fig. 1).

### 3.2 Study characteristics

Table 1 describes the studies’ characteristics. Of the 40 included articles, 21 studied motivational interviewing (MI) or motivational enhancement therapy (MET), 8 studied cognitive behavioral therapy (CBT) or social learning therapy (SLT), 9 studied incentive-based contingency management, 1 studied mindfulness and 1 studied hypnosis. To summarize, 70% (n = 28/40) of the included articles are published in the last decade, 60% (n = 24/40) are conducted in the USA and 75% (n = 30/40) are randomized controlled trials. Overall, the mean study quality score based upon the ErasmusAGE quality assessment was 6.8 (range 5–9).

**Table 1:** Characteristics of the included studies.

Author	Year	Country	Study design	Participants	Sample size	Time period	Lifestyle behavior	Intervention	Control	QS
Ásbjörnsdóttir et al.	2019	Denmark	Cohort study	Women < 20 weeks pregnant, aged ≥ 18 years, with type II diabetes	N = 219 I = 116 C = 103	August 2015 to February 2018	Dietary intake	MI	Standard care	8
Bogaerts et al.	2013	Belgium	RCT	Women ≤ 15 weeks pregnant, with a BMI ≥ 29 kg/m^2^	N = 205 I_1_ = 64 I_2_ = 78 C = 63	March 2008 to April 2011	Dietary intake Physical activity	I_1_ = Brochure I_2_ = Brochure + MI	Standard care	7
Claesson et al.	2008	Sweden	Case-control study	Pregnant women with a BMI ≥ 30 kg/m^2^	N = 348 I = 155 C = 193	November 2003 to December 2005	Dietary intake Physical activity	MI	Standard care	7
Epel et al.	2019	USA	Trial	Women 12–19 weeks pregnant	N = 215 I = 110 C = 105	August 2011 to June 2013	Dietary intake Physical activity	Mindfulness	Standard care	7
Ershoff et al.	1999	USA	RCT	Women ≤ 26 weeks pregnant, aged ≥ 18 years, smoking ≥ 7 cigarettes/week	N = 332 I_1_ = 111 I_2_ = 120 I_3_ = 101	November 1996 to June 1997	Smoking	I_1_ = Booklet I_2_ = Booklet + IVR I_3_ = Booklet + MI	No control situation	8
Farhodimoghadam et al.	2020	Iran	RCT	Women 20–24 weeks pregnant, aged > 19 years	N = 70 I = 35 C = 35	February to June 2017	Dietary intake Physical activity	CBT	Standard care	6
Farhodimoghadam et al.	2019	Iran	RCT	Women 20–24 weeks pregnant, aged > 19 years	N = 66 I = 33 C = 33	February to June 2017	Dietary intake Physical activity	CBT	Standard care	5
Gesell et al.	2015	USA	RCT	Women 10–28 weeks pregnant	N = 135 I = 68 C = 67	January to April 2011	Dietary intake Physical activity	CBT/SLT	Standard care	6
Glover et al.	2015	NZ	RCT	Māori women 2–30 weeks pregnant, aged ≥ 16 years, smoking daily	N = 24 I_1_ = 8 I_2_ = 8 C = 8	December 2012 to June 2013	Smoking	CM; incentives I_1_ = Vouchers I_2_ = Products	Standard care	5
Handmaker et al.	1999	USA	RCT	Pregnant women consuming ≥ 1 alcoholic drink in the past month	N = 42 I = 21 C = 21	Not described	Alcohol consumption	MI	Informational letters	5
Harrison et al.	2013	NZ	RCT	Women 12–15 weeks pregnant with a BMI ≥ 25 kg/m^2^ or a BMI ≥ 23 kg/m^2^ with a Polynesian, Asian or African ethnicity, and with an increased risk for developing GDM	N = 228 I = 121 C = 107	Not described	Dietary intake Physical activity	SLT	ECC	8
Haug et al.	2004	USA	RCT	Women ≤ 26 weeks pregnant opioid dependent receiving methadone pharmacotherapy, smoking ≥ 5 cigarettes/day	N = 63 I = 30 C = 33	Not described	Smoking	MET	Standard care	7
Hayes et al.	2013	Ireland	Controlled before-and-after-study	Pregnant women, aged 16–40 years, smoking	N = 1,000 I = 500 C = 500	June 2004 to June 2007	Smoking	MI	Standard care	6
Heil et al.	2008	USA	RCT	Women ≤ 20 weeks pregnant, smoking	N = 82 I = 40 C = 42	Not described	Smoking	CM; incentives Vouchers	Non-contingent vouchers	5
Higgins et al.	2014	USA	RCT	Women ≤ 25 weeks pregnant, smoking (within the past 7 days)	N = 130 I_1_ = 44 I_2_ = 44 C = 42	December 2006 to June 2012	Smoking	CM; incentives I_1_ = Usual vouchers I_2_ = Revised vouchers	Non-contingent vouchers	8
Jones et al.	2011	USA	RCT	Women ≤ 35 weeks pregnant, aged ≥ 18 years, with opioid and/or cocaine substance use disorder	N = 89 I = 47 C = 42	September 2003 to November 2007	Drug use	RBT	Standard care	7
Jones et al.	2001	USA	RCT	Pregnant women aged ≥ 18 years opiate dependent with cocaine use, meeting the requirements for methadone-maintenance treatment	N = 80 I = 44 C = 36	October 1996 and August 1997	Drug use	CM; incentives	Standard care	5
Joya et al.	2016	Spain	RCT	Pregnant women with a maternal hair length of ≥ 9 cm at delivery (hair growth 1 cm/month)	N = 168 I = 83 C = 85	2014	Alcohol consumption	MI	ECC	7
Karlsen et al.	2013	Denmark	Retrospective study	Women referred to a fertility center in Denmark with a BMI ≥ 30 kg/m^2^	N = 187 I = 110 C = 73	2006 to 2011	Dietary intake Physical activity	MI	MI by phone/e-mail or no MI	5
Krukowski et al.	2017	USA	Cohort study	Women planning pregnancy or < 10 weeks pregnant, aged ≥ 21 years, with a BMI 18.5–35 kg/m^2^	N = 458 I = 230 C = 228	2011 to 2014	Dietary intake Physical activity	MI	Standard care	6
Kurti et al.	2020	USA	Trial	Women < 25 weeks pregnant, aged ≥ 18 years, smoking (within the past 7 days), with a smartphone	N = 60 I = 30 C = 30	Time period	Smoking	CM; incentives	Standard cessation care	6
Mojahed et al.	2018	Iran	RCT	Pregnant women, consuming hookah	N = 140 I = 70 C = 70	2017	Smoking	MI	Standard care	7
Osterman et al.	2014	USA	RCT	Women ≤ 36 weeks pregnant, aged 18–44 years, who have consumed alcohol in the previous year	N = 122 I = 62 C = 60	Not described	Alcohol consumption	MI	Standard care	7
Phelan et al.	2018	USA	RCT	Women 9–16 weeks pregnant, aged ≥ 18 years, with a BMI ≥ 25 kg/m^2^	N = 257 I = 129 C = 128	November 2012 to May 2016	Dietary intake Physical activity	SLT with partial meal replacement	Standard care	9
Phelan et al.	2011	USA	RCT	Women 10–16 weeks pregnant, aged ≥ 18 years, with a BMI 19.8–40 kg/m^2^	N = 401 I = 201 C = 200	2006 to 2008	Dietary intake Physical activity	SLT	Standard care	9
Phillips et al.	2019	USA	RCT	Women ≤ 16 weeks pregnant, aged 18–45 years, with a BMI ≥ 25 kg/m^2^	N = 136 I = 65 C = 71	December 2013 to December 2017	Dietary intake Physical activity	CM; incentives	Standard care	7
Poston et al.	2015	UK	RCT	Women 15–18 weeks pregnant, aged > 16 years, with a BMI ≥ 30 kg/m^2^	N = 1,555 I = 782 C = 772	March 2009 to June 2014	Dietary intake Physical activity	CBT	Standard care	7
Rigotti et al.	2006	USA	RCT	Women ≤ 26 weeks pregnant, aged ≥ 18 years, smoking (within the past 7 days)	N = 442 I = 220 C = 222	September 2001 to June 2004	Smoking	Telephone counseling (MI + SLT)	“Best-practice” brief-counseling	8
Smith et al.	2016	USA	RCT	Pregnant women who participated in < 3 sessions of exercise for ≥ 30 min/week for ≥ 6 months before conception, aged 18–45 years	N = 51 I = 26 C = 25	January to September 2013	Dietary intake Physical activity	Web-based CBT	Standard care	8
Stotts et al.	2002	USA	RCT	Women ≤ 20 weeks pregnant, aged ≥ 18 years, smoking ≥ 5 cigarettes/week before conception	N = 269 I = 134 C = 135	Not described	Smoking	MI	Standard cessation care	8
Tappin et al.	2015	UK	RCT	Women < 24 weeks pregnant, aged ≥ 16 years, with an breath CO test result ≥ 7 ppm	N = 609 I = 306 C = 303	December 2011 to February 2013	Smoking	CM; incentives Vouchers	Standard cessation care	7
Tappin et al.	2005	UK	RCT	Women ≤ 24 weeks pregnant, smoking	N = 762 I = 351 C = 411	March 2001 to May 2003	Smoking	MI	Standard cessation care	8
Tuten et al.	2012	USA	RCT	Women ≤ 30 weeks pregnant, aged ≥ 18 years, nicotine dependent or smoking ≥ 10 cigarettes/day	N = 102 I_1_ = 42 I_2_ = 28 C = 32	May 2005 to January 2009	Smoking	I_1_ = CM; incentives I_2_ = non-contingent behavioral incentives	Standard care	6
Tzilos Wernette et al.	2018	USA	RCT	Women < 20 weeks pregnant (unplanned), who endorsed condomless vaginal/anal sex (at least once in the past 30 days), (at risk of) consuming alcohol or using drugs	N = 50 I = 31 C = 19	December 2015 to April 2016	Alcohol consumption Drug use	MI	Computer-delivered assessment	7
Valanis et al.	2001	USA	Cohort study	Pregnant women, smoking (within the past 7 days or within the month before conception but not within the 7 days before clinic registration)	N = 3,907 I = 2,055 C_1_ = 1,028 C_2_ = 824	January 1992 to December 1996	Smoking	MI	C_1_ historical = standard care C_2_ interim = standard care	6
Valbo et al.	1996	Norway	RCT	Women ± 18 weeks pregnant, smoking	N = 158 I = 80 C = 78	January 1992 to June 1993	Smoking	Hypnosis	Standard care	7
Van der Windt et al.	2020	The Netherlands	Before-and-after study	Women planning pregnancy or ≤ 12 weeks pregnant	N = 450	June 2018 to December 2018	Smoking Alcohol consumption Dietary intake Physical activity	MI	Standard care	5
Winhusen et al.	2008	USA	RCT	Pregnant women, aged ≥ 18 years, needing substance abuse treatment	N = 200 I = 102 C = 98	Not described	Alcohol consumption Drug use	MET	Standard care	8
Yonkers et al.	2012	USA	RCT	Women < 28 weeks pregnant, aged ≥ 16 years, consuming alcohol or using an illicit drug (other than opiates) during the 28 days prior to screening or scored ≥ 3 on the modified TWEAK	N = 183 I = 92 C = 91	June 2006 to July 2010	Alcohol consumption Drug use	MET-CBT	Brief advice	6
Zhang et al.	2017	USA	Cohort study	Pregnant women, smoking	N = 12,434 I = 866 C = 11,568	April 2014 to June 2015	Smoking	MI	Standard care	6

Abbreviations: BMI, body mass index; CBT, cognitive behavioral therapy; CM, contingency management; ECC, educational control condition; GDM, gestational diabetes mellitus; IVR, interactive voice response; MET, motivational enhancement therapy; MI, motivational interviewing; NZ, New Zealand; QS, quality score; RBT, reinforcement based treatment; RCT, randomized controlled trial; SLT, social learning therapy; UK; United Kingdom; USA, United States of America.

### 3.3 Synthesis of results

An overview of included psychological therapies, their goal, and key concepts can be retrieved from Table 2.

**Table 2:** Overview of included different psychological therapies in general, their intended goals, and key concepts.

**Type of psychological therapy**	**Characteristics**
Motivational interviewing (MI)(Rubak et al., 2005) and motivational enhancement therapy (MET)(Guydish et al., 2010)	• Counselling style for provoking behavior change by helping clients to explore and resolve ambivalence. • Overall goal: To increase the client's intrinsic motivation for behavior change. • Key concepts: Ambivalence about current behavior is normal and constitutes an important motivational obstacle in behavior change. Ambivalence can be resolved by working with a client's intrinsic motivations and values. • While MI represents a broader therapeutic approach, MET has a strong focus on personalized assessment, feedback, and change plans.
Cognitive behavioral therapy (CBT) and social learning therapy (SLT)(Fabricatore, 2007, Hofmann et al., 2012)	• Class of structured, action-oriented interventions that focuses on identifying and restructuring negative patterns of thought and behavior. • Overall goal: To help the individual enact change in thinking patterns and behaviors, thereby improving quality of life not by changing the circumstances in which the individual lives, but by helping the individual taking control of his or her own perception of and behaviors in those circumstances. • Key concepts: Cognitions impact emotions and subsequent behaviors and it is possible to intentionally modify the manner in which someone responds to events or thoughts. • The core of SLT is to learn new behaviors by observing other people. This therapeutic strategy can be applied in itself, but is often also an element of CBT.
Incentive-based contingency management(Petry, 2011)	• A type of behavioral therapy in which individuals are ‘reinforced’, or rewarded, for evidence of positive behavioral change. • Overall goal: To stimulate positive behavior. • Key concept: Behaviors that are rewarded are more likely to continue and continue with increased frequency, intensity, and duration.
Mindfulness(Kabat-Zinn, 2003)	• The practice of reaching a ‘full awareness that emerges through paying attention on purpose, in the present moment, and nonjudgmentally to the unfolding of experience moment by moment’. • Overall goal: To be in touch with the inner workings of our mental, emotional, and physical processes. • Key concept: Increasing awareness of how personal emotions influence decisions and behaviors, can positively change behavior and attitude to life. Focus is on raising awareness, not on actively tackling undesirable thoughts (in contrast to CBT).
Hypnosis(Gruzelier, 1998)	• Commonly referred to as hypnotherapy, is a trance-like state in which a person has heightened focus and concentration. • Overall goal: To set aside the conscious mind, and suggestions given directly to the subconscious mind, where behavior is programmed, bypassing the critical factor of the conscious mind. • Key concepts: Hypnosis causes a person to actively or voluntarily split their consciousness.

#### 3.3.1 Motivational Interviewing/Motivational enhancement therapy

Twenty-one studies reported the effects of MI or MET on lifestyle behaviors or pregnancy outcomes.

##### 3.3.1.1 Smoking

Nine studies focused on the effectiveness of MI or MET on smoking cessation during pregnancy, of which 4 studies showed positive effects (Mojahed and Navidian, 2018, Rigotti et al., 2006, Valanis et al., 2001, Zhang et al., 2017). The tested interventions comprised of 2–6 sessions including MI/MET, either individually, in a group or by telephone. The length of each session varies widely among studies, between a couple of minutes to 90 minutes. The least intensive intervention, in terms of session length and frequency, was conducted by Valanis et al, who provided sessions of MI that added no more than a few minutes to every regularly scheduled clinical contact (Valanis et al., 2001). A significant difference in rate of self-reported sustained smoking cessation during pregnancy between the two groups was reported (OR = 2.7, CI = 1.2–5.7). In a large trial of Zhang et al, 866 smoking pregnant women received 4 sessions of MI and 11,568 smoking pregnant women received routine prenatal care (Zhang et al., 2017). Results, based on self-reported data, showed that significant fewer cigarettes were smoked in the intervention group (high or low attendance; defined as attending 1–2 session(s) or attending 3–4 sessions) compared with the control group (4.7 versus 6.8 versus 9.7, *P* < .0001). However, the retrospective selected control group existed of women who were eligible for inclusion, but did not participate in the study, which might have induced selection bias.

The 5 studies that did not demonstrate significant effects on smoking cessation all relied on verified smoking biochemically, either by plasma, salivary or urine cotinine testing (Ershoff et al., 2000, Haug et al., 2004, Hayes et al., 2013, Stotts et al., 2002, Tappin et al., 2005). Three out of 5 studies can be characterized as less intensive, since the intervention was provided either by telephone or comprised of only 3–10 min during regular antenatal visits (Ershoff et al., 2000, Hayes et al., 2013, Stotts et al., 2002). However, Tappin et al (2005) tested in a RCT among 762 pregnant women an intensive intervention with 2–5 MI sessions at home and did not show significant differences in biochemically verified smoking cessation between the intervention and control group (Tappin et al., 2005).

##### 3.3.1.2 Dietary intake, gestational weight gain and weight loss before pregnancy

One study using MI focused on dietary intake, specifically, vegetable and fruit intake (van der Windt et al., 2020). The study of van der Windt et al investigated a blended care periconception lifestyle intervention combining a lifestyle counseling session using MI with a 26-weeks eHealth coaching program Smarter Pregnancy for pregnant women or women contemplating pregnancy and their partner. They showed significant improvements in vegetable intake, fruit intake, and folic acid supplement use. The effects of MI or MET on dietary intake in (pre)pregnant women was not investigated by other studies.

Five studies focused on GWG or weight loss before conception, of which 4 studies showed positive results (Bogaerts et al., 2013, Claesson et al., 2008, Karlsen et al., 2013, Krukowski et al., 2017). All studies used a quite intensive MI intervention, varying between 4 group sessions in total throughout pregnancy till weekly invitations throughout pregnancy. Three studies focused on GWG among pregnant women with obesity and showed comparable results (Bogaerts et al., 2013, Claesson et al., 2008, Krukowski et al., 2017). In the intervention of Krukowski et al, for instance, MI sessions every 6 weeks resulted in significant less GWG as compared to the control group (9.0 ± 4.2 versus 13.6 ± 8.0 kg, *P* = .001)(Krukowski et al., 2017). The only included study that did not demonstrate significant effects on GWG was that of Ásbjörnsdóttir et al, which provided women with diabetes type 2 with 2-weekly sessions of MI combined with CBT (Ásbjörnsdóttir et al., 2019). The intervention group needed a higher insulin dose and experienced more often hypoglycemia at the late pregnancy visit compared with the control group. They argued that insulin is a growth factor and both insulin and hypoglycemia stimulate appetite and this may have influenced the effect on the GWG.

##### 3.3.1.3 Alcohol consumption and drug use

The use of MI to reduce alcohol consumption among pregnant women was investigated by 5 RCTs (Handmaker et al., 1999, Joya et al., 2016, Osterman et al., 2014; Tzilos Wernette et al., 2018, Yonkers et al., 2012). None of the studies found a significant decrease in alcohol use. The intervention intensity of 3 of these studies is relatively low and comprised of only 1 session of MI to stop alcohol consumption during pregnancy (Handmaker et al., 1999, Joya et al., 2016, Osterman et al., 2014). However, Yonkers et al provided an intensive intervention of 6 MET-CBT sessions to women consuming alcohol or using an illicit drug and did not demonstrate any significant effects on alcohol or drug abstinence (Yonkers et al., 2012). Since, this population has to deal with multiple problems, it is harder to successfully change behavior.

The effects of MI and MET on decreasing maternal drug use was evaluated, beside above mentioned study of Yonkers et al, by Winhusen et al and Tzilos Wernette et al, of which the latter showed positive results (Tzilos Wernette et al., 2018, Winhusen et al., 2008, Yonkers et al., 2012). This pilot RCT found a significant reduction in self-reported marijuana or alcohol use in the intervention group, who were provided with 2 computer-delivered MI sessions, compared with the control group (54% versus 16%, *P* = .015) (Tzilos Wernette et al., 2018). Winhusen et al performed a comparable study and showed no significant treatment effects on self-reported alcohol and or biochemically verified drug use (Winhusen et al., 2008).

##### 3.3.1.4 Pregnancy outcomes

The intervention group in the study of Zhang et al, aimed at reducing cigarette smoking, showed fewer infants born with low birth weight (LBW) (OR = 0.51, 95% CI = 0.30–0.88)(Zhang et al., 2017). The study conducted by Ásbjörnsdóttir et al demonstrated no significant effects on GWG, however, showed fewer LGA infants in the intervention group compared with the control group, 14% versus 27%, respectively (*P* = .04)(Ásbjörnsdóttir et al., 2019).

Bogaerts et al and Claesson et al, showed no significant effects of the intervention on restricting GWG, and reported no effects on adverse pregnancy outcomes, such as prevalence of gestational diabetes mellitus (GDM), pre-eclampsia (PE) and pregnancy-induced hypertension (PIH), (acute or elective) caesarean section rate, instrumental delivery rate, birth weight, gestational age at delivery (Bogaerts et al., 2013, Claesson et al., 2008). The intervention provided in the study of Yonkers et al showed no significant effects on alcohol and drug abstinence and no difference on LBW prevalence (Yonkers et al., 2012).

#### 3.3.2 Cognitive behavioral Therapy/Social learning therapy

Eight studies, all RCTs, investigated the effects of CBT or SLT on improving dietary intake and psychical activity, and thereby, restricting GWG (Farhodimoghadam et al., 2019, Farhodimoghadam et al., 2020, Gesell et al., 2015, Harrison et al., 2013, Phelan et al., 2011, Phelan et al., 2018, Poston et al., 2015, Smith et al., 2016). The effects of CBT or SLT on dietary intake, smoking, alcohol consumption or drug use were not investigated.

##### 3.3.2.1 Gestational weight gain

Five studies showed positive effects of CBT or SLT on GWG (Farhodimoghadam et al., 2020, Gesell et al., 2015, Harrison et al., 2013, Phelan et al., 2011, Phelan et al., 2018). The interventions evaluated in the RCTs of Gesell et al and Farhodimoghadam et al (2020) were the most intensive, as the intervention group received 12 and 8 CBT sessions, respectively. The intervention performed by Gesell et al resulted in significantly fewer women with a normal weight exceeded IOM recommendations on weight gain during pregnancy in the intervention group compared with the control group (6.7 versus 47.1%, *P* = .036).(Gesell et al., 2015) Farhodimoghadam et al (2020) reported a significant difference in mean score of a questionnaire on healthy behaviors in favor of the intervention group (Farhodimoghadam et al., 2020). However, in another article in which the same study was analyzed, no significant difference in mean weight after the intervention was found between both groups (Farhodimoghadam et al., 2019). Other studies that reported positive effects in their intervention groups are characterized by individual or group face-to-face sessions of generally 60–90 min. Most of the effective interventions included an extensive explanation on recommended dietary intake and physical activity. On the contrary, studies that found no effect of the intervention only included online sessions or applied the key principles of CBT in a less extensive way.

##### 3.3.2.2 Physical activity

Three studies investigated the effects of CBT on physical activity parameters and all showed positive results (Harrison et al., 2013, Poston et al., 2015, Smith et al., 2016). In the studies of Harrison et al and Poston et al, 4 sessions of SLT and 6 sessions of social cognitive theory, respectively, were provided. Harrison et al showed that women in the intervention group retained a 20% higher step count compared to controls (5.203 vs. 4.140 steps/day, *P* < .05). Poston et al showed a median difference in physical activity of 295 min/week (95% CI: 105–485) between the intervention group and control group. Smith et al, who only provided access to an SLT-based website, showed comparable effects on physical activity in women contemplating pregnancy.

##### 3.3.2.3 Pregnancy outcomes

Included studies reported no significant effects of CBT on adverse pregnancy outcomes, including GDM, PIH, PE, preterm birth, LBW, macrosomia, caesarean section rate, fetal anomalies and neonatal death (Gesell et al., 2015, Harrison et al., 2013, Phelan et al., 2018, Poston et al., 2015).

##### 3.3.2.4 Incentive-based contingency management

Nine studies examined the effects of incentive-based contingency management on different lifestyle behaviors and pregnancy outcomes (Glover et al., 2015, Heil et al., 2008, Higgins et al., 2014, Jones et al., 2001, Jones et al., 2011, Kurti et al., 2020, Phillips et al., 2019, Tappin et al., 2015, Tuten et al., 2012).

##### 3.3.2.5 Smoking

Six studies focused on the effects of cigarette smoking cessation and all found similar, positive effects. In these studies, more or less comparable financial incentives were used (Heil et al., 2008, Higgins et al., 2014, Kurti et al., 2020, Tappin et al., 2015, Tuten et al., 2012(Glover et al., 2015)). In the large RCT of Tappin et al (2015), vouchers could be earned up to $400 by women allocated to the intervention group. This study showed higher biochemically verified cessation rates in the intervention group compared with the control group (22.5 versus 8.6%; RR of not smoking at the end of pregnancy = 2.63, *P* < .001)(Tappin et al., 2015). Although, Tappin et al (2015) used the highest incentives of included studies, this did not lead to the largest effect size. Heil et al and Higgins et al performed a RCT and rewarded women in the intervention group with vouchers up to $45 and demonstrated significant higher cessation rates in the intervention group compared with the control group, 41 versus 10%, *P* = .003 and 46 versus 13%, *P* = .007, respectively (Heil et al., 2008, Higgins et al., 2014). Tuten et al used a comparable incentive and concluded that a contingent financial incentive intervention can significantly reduce cigarette smoking among methadone-maintained women (*P* < .0001)(Tuten et al., 2012).

##### 3.3.2.6 Gestational weight gain

One study investigated the effectiveness of a financial incentive-based intervention on the adherence with GWG guidelines and found no significant effects (Phillips et al., 2019). In the study of Phillips et al, pregnant women received an individual session every 2 weeks to inform them, among other things, on the principles of behavioral weight management. Up to $550 could be earned if they not exceeded GWG guidelines.

##### 3.3.2.7 Alcohol consumption

Two studies focused on drug abstinence and tested either a financial incentive-based or a reinforcement-based intervention (Jones et al., 2001, Jones et al., 2011). Jones et al (2001) proved the effectiveness of an escalating voucher incentive schedule to earn a maximum of $70 among pregnant women who were opiate dependent with cocaine use (Jones et al., 2001). This resulted in a significant greater biochemically verified drug-abstinence (opiates and cocaine) between the intervention group and the control group. Jones et al (2011) demonstrated no significant effects on drug abstinence of a reinforcement-based intervention in which positive behavior was not financially rewarded, but with the stay in a woman’s only recovery house and a more individualized treatment (Jones et al., 2011).

##### 3.3.2.8 Pregnancy outcomes

Included studies reported no significant effects of incentive-based contingency management on pregnancy outcomes, including miscarriage, GDM, PIH, PE, preterm birth, LBW, macrosomia, neonatal intensive care unit admission and, primary caesarean section (Heil et al., 2008, Higgins et al., 2014, Jones et al., 2001, Tappin et al., 2015, Tuten et al., 2012).

### 3.4 Mindfulness

#### 3.4.1 Gestational weight gain

One study reported the effects of a mindfulness-based intervention on GWG among pregnant low-income women (Epel et al., 2019). In the RCT of Epel et al, 110 pregnant women in the intervention group received 8 weekly 2-h sessions, 2 “booster” telephone sessions, and 1 post-partum group session. The control group, including 105 pregnant women, attended routine prenatal care. No significant effects were reported between the two groups.

### 3.5 Hypnosis

#### 3.5.1 Smoking

One RCT was performed to observe the effects of hypnosis on smoking cessation among pregnant women (Valbø and Eide, 1996). In this study of Valbø and Eide, the intervention group (n = 52) received 2 sessions in which relaxation techniques together with self-hypnotic methods were introduced to combat craving. The control group attended routine pregnancy care (n = 78). No significant difference in quit rate was obtained between the 2 groups, as it was 10% in both groups.

### 3.6 Relative risk

**Fig. 2:**
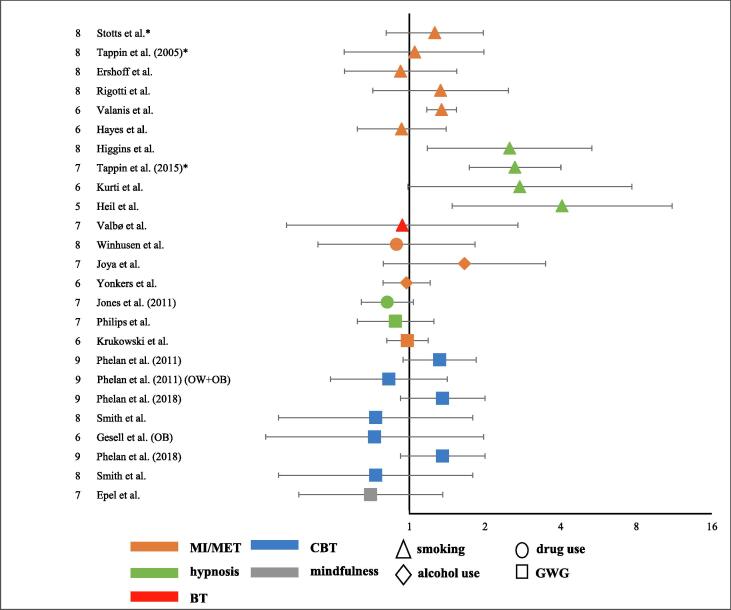
Forest plot of relative risk ratios of included studies on a logarithmic scale QS: ErasmusAGE quality assessment score; GWG: gestational weight gain; MI/MET: motivational interviewing/motivational enhancement therapy; BT: (incentive-based) behavioral therapy; CBT: cognitive behavioral therapy; OW: overweight; OB: obese; *P-value < 0.05.

In Fig. 2 RRs of included studies are displayed. Two studies reported RRs. For 20 studies, we calculated RRs based on numbers provided in the articles. All studies that used incentive-based contingency management for smoking cessation, depicted as green triangles, proved the effectiveness. Moreover, this psychological therapy showed the most uniform results among all reviewed therapies for smoking cessation. The RRs of all other interventions for the improvement of lifestyle behaviors are inconsistent and do not seem to demonstrate their effectiveness convincingly.

## 4 Discussion

Financial incentive-based contingency management and, although less convincingly, MI can reduce smoking behavior among (pre)pregnant women. MI and MET do not show consistent results of effectiveness on improving dietary intake, physical activity, restricting GWG, alcohol consumption and drug use. CBT is not proven effective for improving dietary intake and physical activity. Likewise, incentive-based contingency management is not proven effective for decreasing smoking, drug use or restricting GWG. Additionally, hypnosis and mindfulness do not show positive effects on decreasing smoking and improving dietary intake, respectively. Since effects of psychological interventions on other lifestyle behaviors have not been studied, conclusions on effectiveness cannot be drawn.

### 4.1 Motivational interviewing/motivational enhancement therapy

A large *meta*-analysis of MI versus brief advice or usual care for smoking cessation involving over 16,000 participants yielded a modest but significant increase in quitting (RR 1.26; 95% CI 1.16 to 1.36)(Lindson-Hawley et al., 2015). Contrarily, not all studies included in this review provided compelling evidence for the effectiveness of MI/MET for smoking cessation among (pre)pregnant women. This might be caused by some studies with a low intensive intervention, including telephone counseling or only 3–10 min counseling during regular antenatal visits.

### 4.2 Cognitive behavioral therapy

CBT is a therapeutic approach with the strongest scientific support for the treatment of anxiety disorders, depression, anger control problems, eating disorders, and general stress.(Hofmann et al., 2012) A *meta*-analysis involving 79 trials concluded that CBT is an evidence-based intervention for treating binge eating disorder, the most common eating disorder (Linardon et al., 2017). The goals of CBT for this group is to encourage participants to improve eating patterns and body image by setting goals, self-monitoring, restructuring distorted cognitions and self-perceptions, and managing stress in ways that do not involve food. Since the skills taught in CBT seem to be beneficial for individuals with binge-eating disorder, it is hypothesized that CBT might be an effective treatment modality for obesity as well. However, until now, no conclusive evidence on the effectiveness of CBT for obesity has been provided. The relatively low prevalence, about 5%, of binge eating disorders among obese women, suggests that an adapted approach is required (Kinzl et al., 1999).

Our results on the effectiveness of CBT for weight loss or restricting GWG among (pre)pregnant women correspond to the results for weight loss among the general population.

### 4.3 Incentive-based contingency management

The effectiveness of incentive-based contingency management for lifestyle behavior improvement is widely substantiated, mainly for substance use. A systematic review on smoking cessation among substance users showed that incentive-based contingency management was superior to control arms, with a RR of 2.56 (95% CI: 1.73, 3.78; *P* < .001)(Secades-Villa et al., 2020). This result is comparable to the RRs of incentive-based contingency management for smoking cessation calculated in our systematic review. However, some have argued that any effects are likely to be short-lived as the motivational benefit of rewards will end when the rewards stop (Petry, 2010). None of the studies in our systematic review included a follow-up period after the incentives had stopped.

### 4.4 Mindfulness

Practicing mindfulness could raise an individual's metacognitive awareness of automatic processes associated with craving and substance seeking and using (Li et al., 2017). This awareness may enable an interruption of the cycle of maladapted cognitive, affective, and psychophysiological mechanisms (Garland et al., 2014) (Li et al., 2017, Witkiewitz et al., 2014). A *meta*-analysis of RCTs of mindfulness treatments for substance use showed an OR of −0.33 (95% CI −0.49 - −0.17). Yet, the current review only included one study on the effects of mindfulness on restricting GWG among pregnant women. However, according to the *meta*-analysis, it might be valuable to investigate the effects of mindfulness on substance use among (pre)pregnant women as well (Epel et al., 2019).

### 4.5 Hypnosis

Hypnosis has been suggested as an effective treatment modality to overweight and obesity problems. A recent review and *meta*-analysis concluded that clinicians should view hypnosis as a promising treatment option for obesity, especially when used in conjunction with CBT techniques for weight loss(Milling et al., 2018). However, there is insufficient evidence to determine whether hypnosis is more effective for smoking cessation than other forms of behavioral support or unassisted quitting, according to a review(Barnes et al., 2019).

### 4.6 Recommendations for research and practice

Since we noticed that results differed strongly between studies with self-reported versus objectively measured outcomes, we recommend to include outcomes, as biochemically verified smoking, instead of self-reported smoking behavior. Additionally, we suggest to include an extensive follow up to determine how long intended effects will persist and to define triggers for setback to old habits.

We observed that intensive interventions, consisting of relatively more and longer sessions, were more often effective compared with less intensive interventions. We would, therefore, recommend that more intensive interventions would be preferred over less intensive interventions to increase the effectiveness. However, attention should be paid to attrition rates, since intensive interventions are associated with more participants that withdraw from participation.

In the current review, only one study measured components of dietary intake, while others used GWG as a proxy for dietary intake. Although GWG reflects dietary intake (Itani et al., 2020) and higher GWG is associated with adverse pregnancy outcomes (Sun et al., 2020), wide usage in daily practice and scientific research has been a subject of debate (Abrams et al., 2000). GWG is not a simple sum of the increased maternal body mass, weight of the fetus, placenta and amniotic fluid, but it is a complex biological phenomenon influenced by several changes in maternal physiology and metabolism, such as total body water accretion and fat accretion (National Research, 2010). Therefore, GWG shows considerable variability between individuals, and including GWG in both clinical practice and as an outcome measure in scientific research is doubtful. We encourage a greater focus on dietary intake instead of a sole focus on GWG in clinical and research settings.

Women with lower socioeconomic status more frequently have an unhealthy lifestyle, contributing to greater GWG (O'Brien et al., 2018), and are at greater risk for unintended pregnancies (Iseyemi et al., 2017), and are therefore, less likely to be included in an intervention study to improve lifestyle behaviors in the preconception period. However, women with a lower socioeconomic status may benefit more from lifestyle interventions, if the intervention is delivered in a proper way. Additionally, (pre)pregnant women might be unaware of the necessity and potential health benefits of improving lifestyle behaviors, since they do not experience, in general, any consequences of unhealthy lifestyle behaviors yet. Not only in research settings, but in general practice as well, raising awareness of healthy lifestyle behaviors in the group of (pre)pregnant women is needed.

Socio-economic status and geographical background influence lifestyle behaviors as well as pregnancy outcomes (Kim et al., 2018, Sundquist and Johansson, 1998). Therefore, the provision of individualized lifestyle interventions that take into account women’s socioeconomic status, as well as culture and geographical background(Napier et al., 2014), are the key to successful improvement of lifestyle behaviors, reduction of GWG, and thereby, closing the gap in health inequalities (Terragni et al., 2018).

### 4.7 Strengths and limitations

With the majority of studies being well-designed RCTs, including large sample sizes and objective measurement of outcomes, the quality of included studies was high, with a mean quality score of 6.8 (range: 5–9). The included studies were conducted in different countries, and in a diversity of ethnicities and cultures. In contrast to focusing on one lifestyle behavior and one psychological therapy, the broad scope allowed us to compare the effectiveness of psychological therapies on the improvement of several lifestyle behaviors. However, other factors, such as stress, sleep, and psychological state of mind, are considered as lifestyle behaviors as well (Abe and Abe, 2019). To some degree, this makes our systematic review less comprehensive. However, we preferred to focus on factors not directed related to or representing mental health, since psychological therapies are widely investigated and proven effective for improving those factors.

This systematic review only included studies on cigarette smoking and one study on hookah smoking. Since e-cigarette use increases, among (pre)pregnant women as well, and associated health risk are becoming more evident(Marques et al., 2021), future studies investigating interventions aimed at lifestyle behaviors should include e-cigarette use as well.

Although most studies had a high quality score and included large sample sizes, some studies tested the intervention only on a small group. We tried to highlight these differences by applying the ErasmusAGE quality score that sample size takes into consideration. Additionally, a number of studies were published two decades ago. Since usual care has changed over time, as well as characteristics of, for example, smoking pregnant women(Männistö et al., 2016), comparing recently published studies and studies published longer ago might lead to erroneous conclusions.

At last, due to the large heterogeneity of content and intensity of the psychological therapy interventions, it was not possible to perform a *meta*-analysis. So, we did not have the opportunity to critically evaluate and statistically combine results of comparable studies or trials which could have led to a more precise estimate of the effect sizes and could have increased the generalizability of results of individual studies.

### 4.8 Conclusions

The use of psychological therapies to improve lifestyle behaviors among (pre)pregnant women is relatively new and the emerging scientific proof is promising. Before wide implementation is legitimated, clinical trials should be conducted to study which psychological therapy works for which specific lifestyle behavior and target group, and to study the effects on pregnancy outcomes.

## Declaration of Competing Interest

The authors declare that they have no known competing financial interests or personal relationships that could have appeared to influence the work reported in this paper.

## References

[b0005] Abe M., Abe H. (2019). Lifestyle medicine–An evidence based approach to nutrition, sleep, physical activity, and stress management on health and chronic illness. Personalized Medicine Universe.

[b0010] B. Abrams S.L. Altman K.E. Pickett Pregnancy weight gain: still controversial 71 5 2000 2000 1233S 1241S. 10.1093/ajcn/71.5.1233s 10799396

[b0015] Adams J., Hillier-Brown F.C., Moore H.J., Lake A.A., Araujo-Soares V., White M., Summerbell C. (2016). Searching and synthesising ‘grey literature’and ‘grey information’in public health: critical reflections on three case studies. Systematic reviews.

[b0020] Ásbjörnsdóttir B., Vestgaard M., Ringholm L., Andersen L.L.T., Jensen D.M., Damm P., Mathiesen E.R. (2019). Effect of motivational interviewing on gestational weight gain and fetal growth in pregnant women with type 2 diabetes. BMJ Open Diabetes Res Care.

[b0025] J. Barnes H. McRobbie C.Y. Dong N. Walker J. Hartmann-Boyce Hypnotherapy for smoking cessation 2019 6 2019 10.1002/14651858.CD001008.pub3. 10.1002/14651858.CD001008.pub3 PMC6568235 31198991

[b0030] Blau L.E., Hormes J.M. (2020). Preventing Excess Gestational Weight Gain and Obesity in Pregnancy: the Potential of Targeting Psychological Mechanisms. Curr Obes Rep.

[b0035] Bogaerts A.F.L., Devlieger R., Nuyts E., Witters I., Gyselaers W., Van den Bergh B.R.H. (2013). Effects of lifestyle intervention in obese pregnant women on gestational weight gain and mental health: A randomized controlled trial. Int J Obes.

[b0040] A.R. Brandon Psychosocial interventions for substance use during pregnancy J Perinat Neonatal Nurs 28 169–77 2014 quiz E1–2. 10.1097/JPN.0000000000000041 25062518

[b0045] Brandt C.J., Clemensen J., Nielsen J.B., Søndergaard J. (2018). Drivers for successful long-term lifestyle change, the role of e-health: a qualitative interview study. BMJ Open.

[b0050] Claesson I.M., Sydsjö G., Brynhildsen J., Cedergren M., Jeppsson A., Nyström F., Sydsjö A., Josefsson A. (2008). Weight gain restriction for obese pregnant women: A case-control intervention study. BJOG Int J Obstet Gynaecol.

[b0055] Epel E., Laraia B., Coleman-Phox K., Leung C., Vieten C., Mellin L., Kristeller J.L., Thomas M., Stotland N., Bush N., Lustig R.H., Dallman M., Hecht F.M., Adler N. (2019). Effects of a Mindfulness-Based Intervention on Distress, Weight Gain, and Glucose Control for Pregnant Low-Income Women: A Quasi-Experimental Trial Using the ORBIT Model. Int J Behav Med.

[b0060] Ershoff D.H., Quinn V.P., Boyd N.R., Stern J., Gregory M., Wirtschafter D. (2000). The Kaiser Permanente prenatal smoking cessation trial: when more isn't better, what is enough?. Tob Control.

[b0065] Fabricatore A.N. (2007). Behavior therapy and cognitive-behavioral therapy of obesity: is there a difference?. J. Am. Diet. Assoc..

[b0070] Farhodimoghadam M., Heydarpour S., Salari N., Jaberghaderi N. (2019). The Effect of Cognitive-Behavioural Counselling on Pregnant Women's Weight Gain during Pregnancy: A Randomised Controlled Clinical Trial. Journal of Clinical and Diagnostic Research.

[b0075] Farhodimoghadam M., Heydarpour S., Salari N., Jaberghaderi N. (2020). The Effect of Cognitive-Behavioral Counseling on Lifestyle in Pregnant Women: A Randomized Controlled Clinical Trial. J Med Life.

[b0080] Garland E.L., Manusov E.G., Froeliger B., Kelly A., Williams J.M., Howard M.O. (2014). Mindfulness-oriented recovery enhancement for chronic pain and prescription opioid misuse: results from an early-stage randomized controlled trial. J Consult Clin Psychol.

[b0085] Gesell S.B., Katula J.A., Strickland C., Vitolins M.Z. (2015). Feasibility and Initial Efficacy Evaluation of a Community-Based Cognitive-Behavioral Lifestyle Intervention to Prevent Excessive Weight Gain During Pregnancy in Latina Women. Matern Child Health J.

[b0090] Glover M., Kira A., Walker N., Bauld L. (2015). Using incentives to encourage smoking abstinence among pregnant indigenous women? A feasibility study. Matern Child Health J.

[b0095] Gluckman P.D., Hanson M.A., Cooper C., Thornburg K.L. (2008). Effect of in utero and early-life conditions on adult health and disease. N Engl J Med.

[b0100] Gruzelier J. (1998). A working model of the neurophysiology of hypnosis: A review of evidence. Contemporary Hypnosis.

[b0105] Guydish J., Jessup M., Tajima B., Manser S.T. (2010). Adoption of motivational interviewing and motivational enhancement therapy following clinical trials. J Psychoactive Drugs Suppl.

[b0110] Handmaker N.S., Miller W.R., Manicke M. (1999). Findings of a pilot study of motivational interviewing with pregnant drinkers. J. stud. alcohol.

[b0115] Harrison C.L., Lombard C.B., Strauss B.J., Teede H.J. (2013). Optimizing healthy gestational weight gain in women at high risk of gestational diabetes: A randomized controlled trial. Obesity.

[b0120] Haug N.A., Duffy M., McCaul M.E. (2014). Substance abuse treatment services for pregnant women: psychosocial and behavioral approaches. Obstet Gynecol Clin North Am.

[b0125] Haug N.A., Svikis D.S., DiClemente C. (2004). Motivational enhancement therapy for nicotine dependence in methadone-maintained pregnant women. Psychol Addict Behav.

[b0130] Hayes C.B., Collins C., O'Carroll H., Wyse E., Gunning M., Geary M., Kelleher C.C. (2013). Effectiveness of motivational interviewing in influencing smoking cessation in pregnant and postpartum disadvantaged women. Nicotine Tob Res.

[b0135] S.H. Heil S.T. Higgins I.M. Bernstein L.J. Solomon R.E. Rogers C.S. Thomas G.J. Badger M.E. Lynch Effects of voucher-based incentives on abstinence from cigarette smoking and fetal growth among pregnant women 103 6 2008 1009 1018. 10.1111/j.1360-0443.2008.02237.x PMC2731575 18482424

[b0140] Higgins S.T., Washio Y., Lopez A.A., Heil S.H., Solomon L.J., Lynch M.E., Hanson J.D., Higgins T.M., Skelly J.M., Redner R., Bernstein I.M. (2014). Examining two different schedules of financial incentives for smoking cessation among pregnant women. Prev Med.

[b0145] Hill B., Skouteris H., Boyle J.A., Bailey C., Walker R., Thangaratinam S., Sundseth H., Stephenson J., Steegers E., Redman L.M., Montanaro C., Lim S., Jorgensen L., Jack B., Borges A.L.V., Bergmeier H.J., Baxter J.-A., Harrison C.L., Teede H.J. (2020). Health in Preconception, Pregnancy and Postpartum Global Alliance: International Network Pregnancy Priorities for the Prevention of Maternal Obesity and Related Pregnancy and Long-Term Complications. J Clin Med.

[b0150] Hofmann S.G., Asnaani A., Vonk I.J.J., Sawyer A.T., Fang A. (2012). The Efficacy of Cognitive Behavioral Therapy: A Review of Meta-analyses. Cognit Ther Res.

[b0155] Iseyemi A., Zhao Q., McNicholas C., Peipert J.F. (2017). Socioeconomic Status As a Risk Factor for Unintended Pregnancy in the Contraceptive CHOICE Project. Obstet Gynecol.

[b0160] Itani L., Radwan H., Hashim M., Hasan H., Obaid R.S., Ghazal H.A., Al Hilali M., Rayess R., Mohamed H.J.J., Hamadeh R., Al Rifai H., Naja F. (2020). Dietary patterns and their associations with gestational weight gain in the United Arab Emirates: results from the MISC cohort. Nutr J.

[b0165] Jones H.E., Haug N., Silverman K., Stitzer M., Svikis D. (2001). The effectiveness of incentives in enhancing treatment attendance and drug abstinence in methadone-maintained pregnant women. Drug Alcohol Depend.

[b0170] H.E. Jones K.E. O’Grady M. Tuten Reinforcement-based treatment improves the maternal treatment and neonatal outcomes of pregnant patients enrolled in comprehensive care treatment 20 3 2011 196 204. 10.1111/j.1521-0391.2011.00119.x PMC3084548 21477047

[b0175] Joya X., Mazarico E., Ramis J., Pacifici R., Salat-Batlle J., Mortali C., García-Algar O., Pichini S. (2016). Segmental hair analysis to assess effectiveness of single-session motivational intervention to stop ethanol use during pregnancy. Drug Alcohol Depend.

[b0180] Kabat-Zinn J. (2003). Mindfulness-based interventions in context: past, present, and future. Clinical psychology: Science and practice.

[b0185] Karlsen K., Humaidan P., Sørensen L.H., Alsbjerg B., Ravn P. (2013). Motivational interviewing: a part of the weight loss program for overweight and obese women prior to fertility treatment. Gynecol Endocrinol.

[b0190] Kim M.K., Lee S.M., Bae S.H., Kim H.J., Lim N.G., Yoon S.J., Lee J.Y., Jo M.W. (2018). Socioeconomic status can affect pregnancy outcomes and complications, even with a universal healthcare system. Int J Equity Health.

[b0195] Kinzl J.F., Traweger C., Trefalt E., Mangweth B., Biebl W. (1999). Binge eating disorder in females: a population-based investigation. Int J Eat Disord.

[b0200] Krukowski R.A., West D., DiCarlo M., Shankar K., Cleves M.A., Tedford E., Andres A. (2017). A Behavioral Intervention to Reduce Excessive Gestational Weight Gain. Matern Child Health J.

[b0205] Kurti A.N., Tang K., Bolivar H.A., Evemy C., Medina N., Skelly J., Nighbor T., Higgins S.T. (2020). Smartphone-based financial incentives to promote smoking cessation during pregnancy: A pilot study. Prev Med..

[b0210] Lachman M.E., Lipsitz L., Lubben J., Castaneda-Sceppa C., Jette A.M. (2018). When Adults Don't Exercise: Behavioral Strategies to Increase Physical Activity in Sedentary Middle-Aged and Older Adults. Innov. Aging.

[b0215] Li W., Howard M.O., Garland E.L., McGovern P., Lazar M. (2017). Mindfulness treatment for substance misuse: A systematic review and meta-analysis. J Subst Abuse Treat.

[b0220] Li Y., Pan A.n., Wang D.D., Liu X., Dhana K., Franco O.H., Kaptoge S., Di Angelantonio E., Stampfer M., Willett W.C., Hu F.B. (2018). Impact of Healthy Lifestyle Factors on Life Expectancies in the US Population. Circulation.

[b0225] Y. Li J. Schoufour D.D. Wang K. Dhana A.n. Pan X. Liu M. Song G. Liu H.J. Shin Q.i. Sun L. Al-Shaar M. Wang E.B. Rimm E. Hertzmark M.J. Stampfer W.C. Willett O.H. Franco F.B. Hu Healthy lifestyle and life expectancy free of cancer, cardiovascular disease, and type 2 diabetes: prospective cohort study l6669 10.1136/bmj.l6669. 10.1136/bmj.l6669 PMC7190036 31915124

[b0230] Liberati A., Altman D.G., Tetzlaff J., Mulrow C., Gøtzsche P.C., Ioannidis J.P.A., Clarke M., Devereaux P.J., Kleijnen J., Moher D. (2009). The PRISMA statement for reporting systematic reviews and meta-analyses of studies that evaluate health care interventions: explanation and elaboration. J. Clin. Epidemiol..

[b0235] Linardon J., Wade T.D., de la Piedad Garcia X., Brennan L. (2017). The efficacy of cognitive-behavioral therapy for eating disorders: A systematic review and meta-analysis. J Consult Clin Psychol.

[b0240] Lindson-Hawley N., Thompson T.P., Begh R. (2015). Motivational interviewing for smoking cessation. Cochrane Database Syst.

[b0245] Loef M., Walach H. (2012). The combined effects of healthy lifestyle behaviors on all cause mortality: a systematic review and meta-analysis. Prev Med.

[b0250] Männistö T., Bloigu A., Heino A., Gissler M., Surcel H.M. (2016). Changes in objectively measured smoking in pregnancy by time and legislative changes in Finland: a retrospective cohort study. BMJ open.

[b0255] Marques P., Piqueras L., Sanz M.-J. (2021). An updated overview of e-cigarette impact on human health. Respir. Res..

[b0260] Milling L.S., Gover M.C., Moriarty C.L. (2018). The effectiveness of hypnosis as an intervention for obesity: A meta-analytic review. Psychology of Consciousness: Theory, Research, and Practice.

[b0265] Mojahed K., Navidian A. (2018). The Effect of Motivational Interviewing on Craving and Dependence on Hookah in Suburban Pregnant Women in South East of Iran. Issues Ment Health Nurs.

[b0270] Napier A.D., Ancarno C., Butler B., Calabrese J., Chater A., Chatterjee H., Guesnet F., Horne R., Jacyna S., Jadhav S., Macdonald A., Neuendorf U., Parkhurst A., Reynolds R., Scambler G., Shamdasani S., Smith S.Z., Stougaard-Nielsen J., Thomson L., Tyler N., Volkmann A.-M., Walker T., Watson J., de C Williams A.C., Willott C., Wilson J., Woolf K. (2014). Culture and health. Lancet.

[b0275] National Research, C., 2010. Weight gain during pregnancy: reexamining the guidelines. 20669500

[b0280] E.C. O'Brien G. Alberdi F.M. McAuliffe The influence of socioeconomic status on gestational weight gain: a systematic review 40 1 2018 2018 41 55. 10.1093/pubmed/fdx038 28398550

[b0285] Osterman R.L., Carle A.C., Ammerman R.T., Gates D. (2014). Single-session motivational intervention to decrease alcohol use during pregnancy. J. Subst. Abuse Treat..

[b0290] Oteng-Ntim E., Varma R., Croker H., Poston L., Doyle P. (2012). Lifestyle interventions for overweight and obese pregnant women to improve pregnancy outcome: systematic review and meta-analysis. BMC Med.

[b0295] M.J. Page J.E. McKenzie P.M. Bossuyt I. Boutron T.C. Hoffmann C.D. Mulrow L. Shamseer J.M. Tetzlaff E.A. Akl S.E. Brennan R. Chou J. Glanville J.M. Grimshaw A. Hróbjartsson M.M. Lalu T. Li E.W. Loder E. Mayo-Wilson S. McDonald L.A. McGuinness L.A. Stewart J. Thomas A.C. Tricco V.A. Welch P. Whiting D. Moher n71 10.1136/bmj.n71.

[b0300] Peeters A., Barendregt J.J., Willekens F., Mackenbach J.P., Mamun A.A., Bonneux L. (2003). Obesity in adulthood and its consequences for life expectancy: a life-table analysis. Ann Intern Med.

[b0305] NANCY.M. PETRY Contingency management treatments: controversies and challenges 105 9 2010 1507 1509. 10.1111/j.1360-0443.2009.02879.x PMC3049167 20707772

[b0310] Petry N.M. (2011). Contingency management: what it is and why psychiatrists should want to use it. Psychiatrist.

[b0315] S. Phelan M.G. Phipps B. Abrams F. Darroch A. Schaffner R.R. Wing Randomized trial of a behavioral intervention to prevent excessive gestational weight gain: The Fit for delivery study 93 4 2011 2011 772 779. 10.3945/ajcn.110.005306 PMC3057546 21310836

[b0320] S. Phelan R.R. Wing A. Brannen A. McHugh T.A. Hagobian A. Schaffner E. Jelalian C.N. Hart T.O. Scholl K. Munoz-Christian E. Yin M.G. Phipps S. Keadle B. Abrams Randomized controlled clinical trial of behavioral lifestyle intervention with partial meal replacement to reduce excessive gestational weight gain 107 2 2018 2018 183 194. 10.1093/ajcn/nqx043 PMC6455030 29529157

[b0325] Phillips J.K., Skelly J.M., Roberts L.M., Bernstein I.M., Higgins S.T. (2019). Combined financial incentives and behavioral weight management to enhance adherence with gestational weight gain guidelines: a randomized controlled trial. American J Obstet Gynecol MFM.

[b0330] Poston L., Bell R., Croker H., Flynn A.C., Godfrey K.M., Goff L., Hayes L., Khazaezadeh N., Nelson S.M., Oteng-Ntim E., Pasupathy D., Patel N., Robson S.C., Sandall J., Sanders T.A.B., Sattar N., Seed P.T., Wardle J., Whitworth M.K., Briley A.L. (2015). Effect of a behavioural intervention in obese pregnant women (the UPBEAT study): A multicentre, randomised controlled trial. Lancet Diabetes Endocrinol.

[b0335] Rigotti N.A., Park E.R., Regan S., Chang Y., Perry K., Loudin B., Quinn V. (2006). Efficacy of telephone counseling for pregnant smokers: a randomized controlled trial. Obstet Gynecol.

[b0340] Rubak S., Sandbaek A., Lauritzen T., Christensen B. (2005). Motivational interviewing: a systematic review and meta-analysis. Br J Gen Pract.

[b0345] Secades-Villa R., Aonso-Diego G., García-Pérez Á., González-Roz A. (2020). Effectiveness of contingency management for smoking cessation in substance users: A systematic review and meta-analysis. J Consult Clin Psychol.

[b0350] Smith K., Lanningham-Foster L., Welch A., Campbell C. (2016). Web-Based Behavioral Intervention Increases Maternal Exercise but Does Not Prevent Excessive Gestational Weight Gain in Previously Sedentary Women. J Phys Act Health.

[b0355] Stotts A.L., DiClemente C.C., Dolan-Mullen P. (2002). One-to-one: a motivational intervention for resistant pregnant smokers. Addict Behav.

[b0360] Sun Y., Shen Z., Zhan Y., Wang Y., Ma S., Zhang S., Liu J., Wu S., Feng Y., Chen Y., Cai S., Shi Y., Ma L., Jiang Y.u. (2020). Effects of pre-pregnancy body mass index and gestational weight gain on maternal and infant complications. BMC Pregnancy Childbirth.

[b0365] Sundquist J., Johansson S.-E. (1998). The influence of socioeconomic status, ethnicity and lifestyle on body mass index in a longitudinal study. Int J Epidemiol.

[b0370] D. Tappin L. Bauld D. Purves K. Boyd L. Sinclair S. MacAskill J. McKell B. Friel A. McConnachie L. de Caestecker C. Tannahill A. Radley T. Coleman Financial incentives for smoking cessation in pregnancy: Randomised controlled trial BMJ 350 jan27 4 2015 h134 h134. 10.1136/bmj.h134 25627664

[b0375] Tappin D.M., Lumsden M.A., Gilmour W.H., Crawford F., McIntyre D., Stone D.H., Webber R., MacIndoe S., Mohammed E. (2005). Randomised controlled trial of home based motivational interviewing by midwives to help pregnant smokers quit or cut down. Br Med J.

[b0380] Terragni L., Beune E., Stronks K., Davidson E., Qureshi S., Kumar B., Diaz E. (2018). Developing culturally adapted lifestyle interventions for South Asian migrant populations: a qualitative study of the key success factors and main challenges. Public Health.

[b0385] Thomas B.H., Ciliska D., Dobbins M., Micucci S. (2004). A process for systematically reviewing the literature: providing the research evidence for public health nursing interventions. Worldviews Evid Based Nurs.

[b0390] Tuten M., Fitzsimons H., Chisolm M.S., Nuzzo P.A., Jones H.E. (2012). Contingent incentives reduce cigarette smoking among pregnant, methadone-maintained women: results of an initial feasibility and efficacy randomized clinical trial. Addiction.

[b0395] Wernette G.T., Plegue M., Kahler C.W., Sen A., Zlotnick C. (2018). A Pilot Randomized Controlled Trial of a Computer-Delivered Brief Intervention for Substance Use and Risky Sex During Pregnancy. J Womens Health (Larchmt).

[b0400] Valanis B., Lichtenstein E., Mullooly J.P., Labuhn K., Brody K., Severson H.H., Stevens N. (2001). Maternal smoking cessation and relapse prevention during health care visits. Am J Prev Med.

[b0405] Valbø A., Eide T. (1996). Smoking cessation in pregnancy: the effect of hypnosis in a randomized study. Addict Behav.

[b0410] van der Windt M., van der Kleij R.M., Snoek K.M., Willemsen S.P., Dykgraaf R.H.M., Laven J.S.E., Schoenmakers S., Steegers-Theunissen R.P.M. (2020). Impact of a Blended Periconception Lifestyle Care Approach on Lifestyle Behaviors: Before-and-After Study. J Med Internet Res.

[b0415] Van Dijk M.R., Huijgen N.A., Willemsen S.P., Laven J.SE., Steegers E.AP., Steegers-Theunissen R.PM. (2016). Impact of an mHealth Platform for Pregnancy on Nutrition and Lifestyle of the Reproductive Population: A Survey. JMIR Mhealth Uhealth.

[b0420] Winhusen T., Kropp F., Babcock D., Hague D., Erickson S.J., Renz C., Rau L., Lewis D., Leimberger J., Somoza E. (2008). Motivational enhancement therapy to improve treatment utilization and outcome in pregnant substance users. J Subst Abuse Treat.

[b0425] Witkiewitz K., Bowen S., Harrop E.N., Douglas H., Enkema M., Sedgwick C. (2014). Mindfulness-based treatment to prevent addictive behavior relapse: theoretical models and hypothesized mechanisms of change. Subst Use Misuse.

[b0430] Yonkers K.A., Forray A., Howell H.B., Gotman N., Kershaw T., Rounsaville B.J., Carroll K.M. (2012). Motivational enhancement therapy coupled with cognitive behavioral therapy versus brief advice: A randomized trial for treatment of hazardous substance use in pregnancy and after delivery. Gen Hosp Psychiatry.

[b0435] Zhang X., Devasia R., Czarnecki G., Frechette J., Russell S., Behringer B. (2017). Effects of Incentive-Based Smoking Cessation Program for Pregnant Women on Birth Outcomes. Matern Child Health J.

[b0440] Zhao G., Ford E.S., Tsai J., Li C., Ahluwalia I.B., Pearson W.S., Balluz L.S., Croft J.B. (2012). Trends in health-related behavioral risk factors among pregnant women in the United States: 2001–2009. J Womens Health (Larchmt).

