# Supplementary material for: Effective psychological therapies to improve lifestyle behaviors in (pre)pregnant women: A systematic review
Source: Prev Med Rep. 2021 Nov 9;24:101631. doi: 10.1016/j.pmedr.2021.101631 (PMC8683997; doi:10.1016/j.pmedr.2021.101631)
Supplement: Supplementary data 1 [file mmc1.docx]

Supplemental Table 1A. Description and summary of findings of studies investigating the effect of motivational interviewing or motivational enhancement therapy.

| **QS** | **Author** | **Year** | **Study design** | **Description of intervention(s)** | **Control situation** | **Relevant outcome*** | | **95% CI** |  |
| --- | --- | --- | --- | --- | --- | --- | --- | --- | --- |
| ***Smoking*** | | | | | | | | | |
| 8 | Ershoff et al. | 1999 | RCT | I_1_ = Self-help booklet tailored to smoking patterns, stage of change, and lifestyle of pregnant smokers  I_2_ = I_1_ plus 7 days per week 24 hours per day access to a computerized telephone cessation program based on IVR-technology  I_3_ = I_1_ plus 4-6 proactive telephone sessions (each 10-15 min) of MI performed by nurse educators:  · Semi-structured content through a checklist prompting queries about stage of change, confidence, (dis)advantages of smoking, temptations, high-risk situations and goals  · Personal handwritten postcards from counselors | No control situation | Biochemically verified smoking abstinence at the end of pregnancy (< 80 ng/ml cotinine in urine) | 22.5 (I_1_) vs. 16.7 (I_2_) vs. 20.8% (I_3_) |  | = |
| 8 | Rigotti et al. | 2006 | RCT | 5 telephone sessions (90 min during pregnancy, 15 min post-delivery) of MI and SLT performed by trained counselors:  · Consistent with the 5-step smoking cessation guideline  · Individually tailored to each woman’s readiness to quit and interest in other pregnancy- and health related topics  · Counseling of smokers not ready to quit focused on cognitive strategies to increase readiness to change  · Counseling of smokers ready to quit focused on cognitive-behavioral cessation and relapse-prevention techniques  · After each session a summary letter and targeted written materials | “Best-practice” brief-counseling: 1 telephone session providing brief smoking counseling (≤ 5 min) performed by a trained counselor | Biochemically verified 7-day point-prevalence smoking abstinence at the end of pregnancy (< 20 ng/ml cotinine in saliva) | OR = 1.37 (overall)  **OR = 2.58 (light smokers)**  **OR = 3.02 (quit attempt before enrollment)** | 0.69 to 2.70  **1.1 to 6.1**  **1.15 to 7.94** | =  +  + |
| 8 | Stotts et al. | 2002 | RCT | 2 individual telephone sessions (each 20-30 min) of MI performed by a trained counselor:  · Building motivation for change by eliciting self-motivational statements, exploring ambivalence about change, obtaining a commitment to change and making an individualized plan for smoking reduction or abstinence  · 1 personalized stages of change-based feedback letter  · Reassessing commitment to change, building motivation, and reevaluating the change plan  · Emphasizing the baby’s health and the benefits to the woman | Standard care: routine prenatal care | Biochemically verified smoking cessation at 34 weeks of pregnancy (< 80 ng/ml cotinine in urine) | 32 vs. 34% |  | = |
| 8 | Tappin et al. | 2005 | RCT | 2-5 sessions/home visits (each 30 min) of MI performed by trained midwifes | Standard care: routine health promotion including information on smoking and pregnancy performed by midwifes | Biochemically verified smoking cessation (< 13.7 ng/ml cotinine in serum, < 14.2 ng/ml cotinine in saliva) | RR = 1.05 | 0.55 to 1.98 | = |
| 7 | Haug et al. | 2004 | RCT | 4 sessions of MET performed by MSc research associates:  · Promoting motivation for change by developing rapport by using empathy and reflective listening  · Providing personalized feedback  · Helping to build the woman’s level of motivation by developing a commitment and plan for change during the pregnancy  · Addressing barriers to long-term change, reviewing motivation and discussing ways to regroup or develop new goals for the future | Standard care: advice on reducing tobacco use | Biochemically verified smoking cessation (< 8 ppm CO in breath, < 200 ng/ml cotinine in urine) |  |  | = |
| 7 | Mojahed et al. | 2018 | RCT | 5-6 group sessions of MI (each 60-90 min) twice per week performed by an individual with MSc in Midwifery Counseling:  · According to the Fields’ model^66^ | Standard care:  recommendations and training regarding the health hazard of hookah smoking | Hookah craving (Hookah Craving Test: range 0-100) | **29.18±5.69 vs. 74.45±5.33** |  | + |
|  |  |  |  |  |  | Hookah dependence (questionnaire: range 0-48) | **15.35±5.01 vs. 41.21±5.55** |  | + |
| 6 | Hayes et al. | 2013 | Controlled before-and-after study | 5 brief sessions of MI, performed by hospital and community personnel | Standard care: routine prenatal care | Biochemically verified smoking abstinence at 28-32 weeks of pregnancy (negative colorimetric assay for cotinine in urine) | 8.2 vs. 8.8% |  | = |
| 6 | Valanis et al. | 2001 | Cohort study | Sessions of MI (each a few minutes) performed by trained clinicians:  · Increasing the woman’s quitting motivation and self-efficacy  · Assessing and documenting the woman’s smoking status and readiness to modify her smoking behavior  · Brief stage-specific, smoking-cessation messages and negotiation of actions towards quitting  · Smoking cessation aids (e.g. self-help brochures, tip sheets, stage-specific videos)  · If a woman quit: emphasizing relapse prevention and sending relapse prevention messages after delivery | Standard care: routine prenatal care | Self-reported smoking abstinence during pregnancy | **OR = 2.7** | **1.2 to 5.7** | + |
| 6 | Zhang et al. | 2017 | Cohort study | 4 sessions (each) of MI performed by trained researchers:  · 5A’s approach^70^  · Post-delivery: monthly visits, $25 voucher for diapers each month for up to 12 months if the breath CO testing results were within the normal ranges | Standard care: routine prenatal care | Self-reported number of cigarettes per day in the third trimester | **4.7 (high attendance) vs. 6.8 (low attendance) vs. 9.7** |  | + |
|  |  |  |  |  |  | LBW (< 2,500 g) | **OR = 0.37 (evidence of quitting)** | **0.17 to 0.79** | + |
|  |  |  |  |  |  | Preterm birth (< 37 weeks of pregnancy) | OR = 0.68 (evidence of quitting) | 0.39 to 1.20 | = |
| ***Alcohol consumption*** | | | | | | | | | |
| 7 | Joya et al. | 2016 | RCT | 1 session of MI:  · Providing personalized feedback of risk behavior  · Motivating the woman to change target behaviors  · Decreasing the temptation to engage in risk behavior and increasing confidence to avoid it  · Developing change plans  · Encouraging the woman to attend the contraceptive counseling visit | ECC: advices for eating, drinking, health screenings, family planning, prevention of sexually transmitted infections and exercise recommendations | Biochemically verified ethanol abstinence (< 7 pg EtG/mg hair) | 46.4 vs. 28 (repeated moderate ethanol consumption)  20 vs. 0 (excessive chronic consumption) |  | =  = |
| 7 | Osterman et al. | 2014 | RCT | 1 session (30 min) of MI performed by a trained researcher:  · Establishing empathy  · Developing discrepancy  · Rolling with resistance  · Supporting self-efficacy | Standard care: routine prenatal care | Self-reported alcohol use during pregnancy | 0.00±0.00 vs. 0.04±0.29 days/week  0.05±0.30 vs. 0.04±0.269 drinks/day |  | =  = |
| 6 | Yonkers et al. | 2012 | RCT | 6 sessions (each 30 min) of MET-CBT performed by trained research nurses:  · Key components: motivational enhancement, functional analysis, safe sexual behavior, communication skills, relapse prevention, problem solving skills | Brief advice: manualized version of standard interventions offered by obstetrical doctors and nurses | Biochemically verified alcohol/drug abstinence (negative immune-chromatographic test for drugs in urine, negative breath alcohol test) from intake to delivery | OR = 0.77 | 0.32 to 1.84 | = |
|  |  |  |  |  |  | LBW (< 2,500 g) | 10 vs. 20% |  | = |
|  |  |  |  |  |  | Preterm birth (< 37 weeks of pregnancy) | 14 vs. 20% |  | = |
| 5 | Handmaker et al. | 1999 | RCT | 1 session (60 min) of MI performed by a trained researcher:  · Increasing the woman’s perceptions of the health risks to her unborn child associated with her current alcohol consumption by giving feedback on the severity of drinking and showing a chart of fetal development  · Supporting the woman’s perceived ability to change | Letters informing about the potential risks of alcohol consumption during pregnancy and referring to health care providers | Self-reported alcohol abstinence during later pregnancy | 44 vs. 33% |  | = |
| ***Drug use*** | | | | | | | | | |
| 7 | Tzilos Wernette et al. | 2018 | RCT | 2 computer-delivered assessments of MI (initial session 60 min, second ‘booster’ session 15 min):  · Providing training in several relevant skills  · Informed by Information-Motivation-Behavior model | 2 computer-delivered assessments: questions about television show preferences and viewing videos of popular entertainers | Reduction in self-reported alcohol/drug use | **54 vs. 16%** |  | + |
| 8 | Winhusen et al. | 2008 | RCT | 3 sessions (initial session 90-120 min, other sessions 60 min) of MET performed by trained clinicians:  · Developing rapport through the use of open ended | Standard care included: 3 individual sessions (initial | Biochemically verified drug abstinence (negative urine test) | 16.7 vs. 14.3% |  | = |
|  |  |  |  | questions including discussion of the woman’s feelings about her pregnancy, reflective listening and affirming the woman, and exploring the woman’s perceived (dis)advantages of using substances  · Individualized personal feedback report  · Developing a change plan for women who expressed a readiness to change and strengthening commitment to change in women who were not yet ready to change  · Encouraging women to participate in the other treatment services | session 90-120 min, other sessions 60 min), performed by a clinician | Alcohol/drug use | 4.26±8.73 vs. 4.70±8.87 monthly days |  | = |
| ***Dietary intake (and physical activity)*** | | | | | | | | | |
| 8 | Ásbjörnsdóttir et al. | 2019 | Cohort study | Individual one-to-one sessions of CBT and MI at each pregnancy visit every 2 weeks performed by a lifestyle coach:  · Making an action plan for improving dietary behavior  · At each visit achieving one dietary objective: watch portion size, eat mainly carbohydrates of low glycemic index, reduce the intake of carbohydrate of high glycemic index  · Motivating women to watch daily weight changes | Standard care: routine diabetes care | GWG | 9.2±5.8 vs. 10.2±5.8 kg |  | = |
|  |  |  |  |  |  | BW | 3167±659 vs. 3324±636 g |  | = |
|  |  |  |  |  |  | CS | 45 vs. 51% |  | = |
|  |  |  |  |  |  | LGA (BW > P90) | **14 vs. 27%** |  | + |
|  |  |  |  |  |  | Perinatal morbidity | 35 vs. 42% |  | = |
|  |  |  |  |  |  | Perinatal mortality | 1 vs. 1% |  | = |
|  |  |  |  |  |  | Preterm birth (< 37 weeks of pregnancy) | 20 vs. 17% |  | = |
|  |  |  |  |  |  | SGA (BW < P10) | 14 vs. 8% |  | = |
|  |  |  |  |  |  | Shoulder dystocia | 1 vs. 1% |  | = |
| 7 | Bogaerts et al. | 2013 |  | I_1_ = brochure about nutritional advice and physical activity during pregnancy with information to limit excessive GWG  I_2_ = brochure plus 4 group sessions (maximum of 3 women) (each 90-120 min) of MI performed by a trained midwife:  · Focusing on the relation between energy intake and energy expenditure based on the active and healthy food pyramid for pregnant women  · Recommendations for a healthy and balanced diet consisting of 50-55% carbohydrate intake, 30-35% fat intake, and 9-11% protein intake  · After every session setting small stepwise goals | Standard care: routine prenatal care | GWG | **9.5 (I_1_) and 10.6 (I_2_) vs. 13.5 kg** |  | + |
|  |  |  |  |  |  | GDM | 12.1(I_1_) vs. 11.8 (I_2_) vs. 11.1% |  | = |
|  |  |  |  |  |  | PE | 2.3 (I_1_) vs. 2.7 (I_2_) vs. 6.3% |  | = |
|  |  |  |  |  |  | PIH | 19.3 (I_1_) vs. 10.8 (I_2_) vs. 9.5% |  | = |
| 7 | Claesson et al. | 2008 | Case-control study | Individual sessions (each 30 min) of MI every week performed by a trained midwife:  · Weight control and supportive talks  · Invitation to an aerobics class designed for obese women | Standard care: routine prenatal care | GWG | **7.52±15.40 vs. 9.78±16.25 kg** |  | = |
|  |  |  |  |  |  | Acute CS | 14.3 vs. 15.8% |  | = |
|  |  |  |  |  |  | BW | 3688.0±680.7 vs. 3678.9±571.3 g |  | = |
|  |  |  |  |  |  | Elective CS | 9.7 vs. 5.2% |  | = |
|  |  |  |  |  |  | GA at delivery | 39.3±2.11 vs. 39.3±2.05 weeks |  | = |
|  |  |  |  |  |  | Instrumental delivery | 10 vs. 9.8% |  | = |
| 6 | Krukowski et al. | 2017 | Cohort study | Sessions (initial session 20-30 min, other sessions 10-20 min) of MI every 6 weeks performed by a trained individual:  · Clear GWG goals for the entire pregnancy and weekly goals, plotting GWG at each intervention session, daily self-weighing, goal setting  · Health journal  · Visual feedback by plotting the total GWG on a graph  · If women gained in excess of the IOM guidelines the intervention was intensified: daily dietary self-monitoring, weekly sessions of motivational interviewing, relevant handouts on various topics | Standard care: routine prenatal care | GWG | **12.7±2.7 vs. 14.2±4.9 kg (normal weight)**  12.4±4.9 vs. 14.0±5.7 kg (overweight)  **9.0±4.2 vs. 13.6±8.0 kg (obese)** |  | +  =  + |
|  |  |  |  |  |  | GWG in excess of the IOM weight gain guidelines | 52.2 vs. 51.1% |  | = |
| 5 | Karlsen et al. | 2013 | Retrospective study | Sessions of MI (face-to-face) performed by a trained nurse:  · Based on trust and confidence with special reference to create a motivational atmosphere  · Promoting self-confidence and induction of initiatives for changes  · Recognizing, supporting and encouraging the woman to set up goals | Sessions of MI by phone/e-mail or no sessions of MI | Decrease in BMI | **3.3 vs. 2.6 kg/m^2^** |  | + |
|  |  |  |  |  |  | Weight loss | **9.3 vs. 7.3 kg** |  | + |
| 5 | Van der Windt et al. | 2020 | Before-and-after study | Blended care approach: 1 face-to-face session of MI performed by a medical doctor at the outpatient clinic ‘Healthy Pregnancy’ and a 6-month online eHealth program ‘Smarter Pregnancy’  ‘Healthy Pregnancy’:  · Tailored lifestyle advice  · Providing possible options to alter lifestyle  ‘Smarter Pregnancy’:  · Personalized coaching on the most prevalent inadequate nutrition and lifestyle behaviors  · Up to 3 short motivating and supporting messages per week by email (e.g. vouchers, seasonal recipes, personalized tips and recommendations)  · Every 6 weeks additional questions addressing behavior, diet and pregnancy status, to monitor lifestyle changes  · Personal page with additional modules to encourage the performance of physical activity, increase compliance with hospital appointments and optimize medication adherence | Standard care: routine prenatal care | Tobacco use | 0.15 vs. 0.20% |  | = |
|  |  |  |  |  |  | Alcohol consumption | **0.19 vs. 0.25%** |  | + |
|  |  |  |  |  |  | Folic acid intake | **1 vs. 0.97%** |  | + |
|  |  |  |  |  |  | Fruit intake | **2.2 vs. 1.8 pieces/day** |  | + |
|  |  |  |  |  |  | Vegetable intake | **165 vs. 151 g/day** |  | + |
|  |  |  |  |  |  |  |  |  |  |

Abbreviations: CI, confidence interval; CBT, cognitive behavioral therapy; CS, cesarean section; ECC, educational control condition; EtG, ethyl glucuronide;

GA, gestational age; GDM, gestational diabetes mellitus; IOM, Institute of Medicine; IOL, induction of labor; IVR, interactive voice response; LBW, low birth weight; LGA, large for gestational age; MET, motivational enhancement therapy; MI, motivational interviewing; MSc, Master of Science; NNT, number needed to treat; OR, odds ratio; P10, 10^th^ percentile; P90, 90^th^ percentile; PE, preeclampsia; PIH, pregnancy-induced hypertension; RCT, randomized controlled trial; RR, relative risk; SEC, standard ethanol content; QS, quality score. *Relevant outcomes are presented as ratios, percentages, mean difference or as mean±SD.

Supplemental Table 1B. Description and summary of findings of studies investigating the effect of cognitive behavioral therapy or social learning therapy.

| **QS** | **Author** | **Year** | **Study design** | **Description of intervention(s)** | **Control situation** | **Relevant outcome*** | | **95% CI** |  |
| --- | --- | --- | --- | --- | --- | --- | --- | --- | --- |
| ***Dietary intake (and physical activity)*** | | | | | | | | | |
| 9 | Phelan et al. | 2018 | RCT | Individual face-to-face sessions (each 20 min) of SLT every 2 weeks until 20 weeks of pregnancy following every month until delivery:  · A structured meal plan, individually tailored to meet each participant’s self-reported dietary needs  · Partial meal replacement plan  · Instructing to replace 2 meals with the provided meal replacement shakes/bars and to consume ≥ 1 meal for regular foods and 2-4 healthy snacks/day  · Encouraging to aim for a goal of 30 min of activity on most days of the week  · A pedometer and a personalized graph of GWG with feedback at each visit  · Reviewing diet and mail replacement records and self-reported intake of the meal replacement at every visit to facilitate problem-solving and adherence  · Other behavioral strategies (daily recording of food, drink, caloric intake and physical activity, stimulus control techniques, problem-solving skills, etc.) | Standard care: routine prenatal care plus 1 face-to-face session (15 min), performed by trained study interventionists and newsletters with general information about pregnancy-related health every 2 months | GWG | **0.33±0.25 vs. 0.39±0.23 kg/week** |  | + |
|  |  |  |  |  |  | GWG in excess of IOM weight gain guidelines | **41.1 vs. 53.9%** |  | + |
|  |  |  |  |  |  | CS | OR = 1.24 | 0.73 to 2.13 | = |
|  |  |  |  |  |  | GDM | OR = 0.90 | 0.46 to 1.96 | = |
|  |  |  |  |  |  | LBW (< 2,500 g) | OR = 1.84 | 0.62 to 5.83 | = |
|  |  |  |  |  |  | Macrosomia (> 4,000 g) | OR = 0.95 | 0.34 to 2.63 | = |
|  |  |  |  |  |  | PE | OR = 1.29 | 0.48 to 3.58 | = |
|  |  |  |  |  |  | PIH | OR = 0.67 | 0.18 to 2.28 | = |
|  |  |  |  |  |  | Preterm birth (< 36 weeks of pregnancy) | OR = 0.76 | 0.18 to 2.98 | = |
| 9 | Phelan et al. | 2011 | RCT | 1 individual session (30 min) of SLT and 3 brief supportive phone calls (each 10-15 min) from a dietitian:  · Discussing appropriate GWG, physical activity (30 min of walking most days of the week), calorie goals (20 kcal/kg)  · Decreasing high fat foods, increasing physical activity, and daily self-monitoring of eating, exercise and weight  · Body-weight scales, food records and pedometers to promote adherence to daily self-monitoring  · Automated postcards prompting healthy eating and exercise habits every week  · A personalized graph of weight gain with feedback at each visit  · Women who were over/under IOM weight gain guidelines during any 1 month interval: additional brief, supportive phone calls (2 calls per month) | Standard care: 1 face-to-face session (15 min), performed by trained study interventionists and newsletters with general information about pregnancy-related health every 2 months | GWG in excess of the IOM weight gain guidelines | **OR = 0.38(normal weight)**  OR = 1.4 (overweight and obese) | 0.20 to 0.87  0.70 to 2.7 | +  = |
|  |  |  |  |  |  | CS |  |  | = |
|  |  |  |  |  |  | GDM |  |  | = |
|  |  |  |  |  |  | LBW (BW < 2,500 g) |  |  | = |
|  |  |  |  |  |  | Macrosomia (BW > 4,000 g) |  |  | = |
|  |  |  |  |  |  | PE |  |  | = |
|  |  |  |  |  |  | PIH | **OR = 0.21 (normal weight)** | **0.05 to 0.96** | + |
|  |  |  |  |  |  | Preterm birth (< 36 weeks of pregnancy) |  |  | = |
| 8 | Harrison et al. | 2013 | RCT | 4 individual sessions of SLT performed by a health coach:  · Supporting and empowering pregnant women to optimize their lifestyle and GWG in an interactive, individualized environment  · Pregnancy-specific dietary advice in addition to simple behavioral change strategies  · Simple behavioral change strategies  · Determining individual goals  · Self-monitoring strategies: pedometer, weight gain charts | 1 brief education session in which GWG was not discussed | GWG at 28 weeks of pregnancy | **6.0±2.8 vs. 6.9±3.3 kg** |  | + |
|  |  |  |  |  |  | Physical activity (pedometer) at 28 weeks of pregnancy | **5.203±3.368 vs. 4.140±2.420 steps/day** |  | + |
|  |  |  |  |  |  | GDM (ADIPS criteria) | 22% (overall) |  | = |
| 7 | Poston et al. | 2015 | RCT | 8 individual or group sessions (each 60 min) of SCT once per week for 8 weeks:  · Addressing approaches to achieve SMART goals  · Advising on self-monitoring, identification, and problem-solving of barriers to behavior change, enlisting social support, providing opportunities for social comparison  · A handbook with information about the intervention and the theory behind it, with recommended foods and recipes, and suggestions for physical activity, DVD of an exercise regimen that was safe for pregnancy, a pedometer and a logbook for recording SMART goals | Standard care: routine prenatal care | GWG | **MD = -0.55 kg** | **-1.08 to -0.02** | + |
|  |  |  |  |  |  | MET | **MD = 295 min/week** | **105 to 485** | + |
|  |  |  |  |  |  | Total energy intake | **MD = -0.70 MJ/day** | **-0.96 to -0.45** | + |
|  |  |  |  |  |  | BW | MD = -27 g | -85 to 31 | = |
|  |  |  |  |  |  | Congenital anomalies | RR = 0.82 | 0.25 to 2.69 | = |
|  |  |  |  |  |  | CS | RR = 0.89 | 0.86 to 1.12 | = |
|  |  |  |  |  |  | GA at delivery | MD = 0.02 weeks | -0.2 to 0.2 | = |
|  |  |  |  |  |  | GDM (IADPSG criteria) | RR = 0.96 | 0.79 to 1.16 | = |
|  |  |  |  |  |  | LGA (BW ≥ P90) | RR = 1.15 | 0.83 to 1.59 | = |
|  |  |  |  |  |  | Neonatal death | RR = 0.98 | 0.14 to 6.97 | = |
|  |  |  |  |  |  | PE | RR = 1.00 | 0.59 to 1.69 | = |
|  |  |  |  |  |  | PPH | RR = 1.19 | 0.91 to 1.54 | = |
|  |  |  |  |  |  | Preterm birth | RR = 0.93 | 0.62 to 1.37 | = |
| 7 | Smith et al. | 2016 | RCT | Access to SCT-based website:  · Including diet and physical activity recommendations, exercise goal-setting modules, problem-solving modules, journal, calendar to track all exercise until delivery, and a community forum to interact with other participants in the intervention group  · Instructions to gradually work up to ≥ 150 min of moderate physical activity per week (in ≥ 10 min bouts) by 19 weeks of pregnancy and sustain at least this amount until delivery  · In-person tutorial with the study coordinator on how to use the website and its features, navigate pertinent information and practice tracking physical activity | Standard care: access to SCT-based website with general diet and physical activity recommendations | GWG | 13.6±5.6 vs. 11.2±5.1 kg |  | = |
|  |  |  |  |  |  | GWG in excess of IOM weight gain guidelines | 68.2 vs. 52.4% |  | = |
|  |  |  |  |  |  | Energy intake at 24-26 weeks of pregnancy | **2,503±703 vs. 1,894±594 kcal/day** |  | - |
|  |  |  |  |  |  | Physical activity at 24-26 weeks of pregnancy | **122±106 vs. 46±48 min/week (20 min bouts)**  **74±70 vs. 14±24 min/week (30 min bouts)** |  | +  + |
| 6 | Gesell et al. | 2015 | RCT | 12 group sessions (90 min each) of CBT/SLT, performed by trained healthcare providers:  · Behavior change strategies focusing on nutrition, exercise, sleep hygiene, coping with stress and anxiety, communication, money and time management, social skills, assertiveness  · Promoting active learning, retention and transfer of knowledge, skills and attitudes by using best practices in instructional design for adults | Standard care: 3 home-visits (each 30 min) performed by hospital interpreters: an infant injury prevention intervention | GWG in excess of IOM guidelines | 27.3 vs. 44.2%  **6.7 vs. 47.1% (normal weight)**  28.6 vs. 40.0% (overweight)  46.7 vs. 45.5% (obese) |  | =  + |
|  |  |  |  |  |  | BW |  |  | = |
|  |  |  |  |  |  | GA at delivery |  |  | = |
| 6 | Farhodimo-  ghadam et al. | 2020 | RCT | 8 group sessions (each 60-90 min) of CBT every week, performed by trained researchers:  · Main subjects: stress-management, sports, nutrition, sleep and rest, social support  · Deep breathing, facing emotional and physical moods, avoiding the stress source/catastrophic thinking  · Verbal improvement, problem-solving, removing obstacles, activating behavior  · Identifying self-thoughts, assessing negative thoughts and replacing them with positive thoughts  · Techniques for dealing with avoidant behavior  · Skills for attracting support and presentation | Standard care: routine prenatal care | Lifestyle score | **151 ±17.72 vs. 139.15±24.77** |  | + |
| 5 | Farhodimo-  ghadam et al. | 2019 | RCT | 8 group sessions (each 60-90 min) of CBT every 2 weeks performed by trained researchers:  · Main subjects: stress-management, sports, nutrition, sleep and rest, social support  · Cognitive restructuring, transformation and correction of distorted thoughts  · Training cognitive behavioral exercises and techniques | Standard care: routine prenatal care | GWG | 74.2±9.47 vs. 73.05±11.51 kg |  | = |
|  |  |  |  |  |  |  |  |  |  |

Abbreviations: ADIPS, Australasian Diabetes in Pregnancy Society; BW, birthweight; CBT, cognitive behavioral therapy; Cesarean section; CI, confidence interval; GA, gestational age; GDM, gestational diabetes mellitus; IADPSG, International Association of Diabetes and Pregnancy Study Groups; IOM; Institute of Medicine; LBW, low birthweight; LGA, large for gestational age; MD, mean difference; OR, odds ratio; P10, 10^th^ percentile; P90, 90^th^ percentile; PE, preeclampsia; PIH, pregnancy induced hypertension; PPH, postpartum hemorrhage; RR, relative risk; SMART, specific, measurable, achievable, relevant, time-specific; SCT, social cognitive therapy; SLT, social learning therapy. *Relevant outcomes are presented as ratios, percentages, mean difference or as mean±SD.

Supplemental Table 1C. Description and summary of findings of studies investigating the effect of contingency management.

| **QS** | **Author** | **Year** | **Study design** | **Description of intervention(s)** | **Control situation** | **Relevant outcome*** | | **95% CI** |  |
| --- | --- | --- | --- | --- | --- | --- | --- | --- | --- |
| ***Smoking*** | | | | | | | | | |
| 8 | Higgins et al. | 2014 | RCT | I_1_ = Usual vouchers:  · $6.25 voucher upon submitting breath CO tests ≤ 6 ppm during the initial 5 days and urine-cotinine levels ≤ 80 ng/ml beginning in week 2 of abstinence monitoring  · $1.25 increase of voucher per consecutive negative tests (maximum: $45)  · Positive test results or missed visit set the voucher value back to the original low value, but 2 consecutive negative tests restored the value to the pre-reset level  I_2_ = Revised vouchers:  · $18.75 voucher upon submitting breath CO tests ≤ 4 ppm during the initial 5 days  · $3.75 increase of voucher per consecutive negative tests (maximum: $33.75)  · $87.50 additional voucher upon testing cotinine negative at the first urine test in week 2  · 5 times $15.50 additional voucher on the second test day of each week during weeks 2-6 if women also had tested negative for smoking at the earlier test conducted that same week  · I_1_ if women submitted breath CO tests > 4 but ≤ 6 ppm during the initial 5 days | Standard care for smoking cessation plus non-contingent vouchers:  · Delivery independent of smoking status  · $15 voucher per visit antepartum  · $20 voucher per visit postpartum | Biochemically verified smoking cessation at late pregnancy (< 6 ppm CO in breath, < 80 ng/ml cotinine in urine) | **45 (I_2_) vs. 36 (I_1_) vs. 18%** |  | + |
|  |  |  |  |  |  | BW | 3284.9±105.8 (I_2_) vs. 3344.8±101.9 (I_1_) vs. 3188.6±105.0 g |  | = |
|  |  |  |  |  |  | GA at delivery | 39.0±0.3 (I_2_) vs. 39.3±0.3 (I_1_) vs. 38.9±0.3 weeks |  | = |
|  |  |  |  |  |  | LBW | 11 (I_2_) vs. 7 (I_1_) vs. 11% |  | = |
|  |  |  |  |  |  | NICU admissions | 8 (I_2_) vs. 2 (I_1_) vs. 11% |  | = |
|  |  |  |  |  |  | Preterm birth | 8 (I_2_) vs. 5 (I_1_) vs. 11% |  | = |
| 7 | Tappin et al. | 2015 | RCT | · £50 shopping voucher for attending a face-to-face appointment and setting a quit date  · £50 shopping voucher upon confirmed quitting at 4 weeks post-quit date  · £100 shopping voucher upon continued validated abstinence after 12 weeks  · £200 shopping voucher upon validated abstinence at 34-38 weeks of pregnancy | Standard cessation care 1 face-to-face session to discuss smoking and cessation, and for women who attended and set a quit date, the offer of free nicotine replacement therapy for 10 weeks provided by pharmacy services, and 4 weekly support phone calls | Biochemically verified smoking abstinence at 34-38 weeks of pregnancy (< 14.2 ng/ml cotinine in saliva, < 44.7 ng/mL cotinine in urine) | **RR = 2.63** | **1.73 to 4.01** | + |
|  |  |  |  |  |  | BW | 3,140±600 vs. 3120±590 g |  | = |
|  |  |  |  |  |  | Preterm birth (< 37 weeks of pregnancy) | RR = 1.52 | 0.95 to 2.39 | = |
|  |  |  |  |  |  | Stillbirth/miscarriage | RR = 0.39 | 0.04 to 1.99 | = |
| 6 | Kurti et al. | 2020 | Trial | · $6.25 voucher for the first negative sample  · $1.00 increase of voucher per consecutive negative test (maximum: $33.25)  · Positive or missing samples reset  the value of the incentive to $6.25  · Submitting 2 negative samples following a reset restored the incentive to its value prior to the lapse  · Delivery using the DynamiCare Rewards smartphone app in the form of money loaded onto a PEX debit card following video validation | Standard cessation care: counseling (10 min) based on the 5A’s approach, quit line referral [Fiore et al. 2008] | Biochemically verified smoking abstinence early antepartum (< 6 ppm CO in breath, < 30 ng/ml cotinine in saliva) | **OR = 3.50** | **1.11 to 11.02** | + |
| 6 | Tuten et al. | 2012 | RCT | I_1_ = CAP-standard care plus contingent incentives:  ·For 3 samples per week if the reduction and abstinent targets were met: week 1: any reduction, week 2-4: 10% reduction, week 5-7: 25% reduction, week 8-9: 50% reduction, week 10-11: 75% reduction, week 12 until delivery: abstinence  · $7.50 initial voucher  · $1/day increase for each consecutive target met throughout 12 weeks incentive period to a maximum of $41.50  · If a woman failed to meet the tobacco use reduction target during the 12 week incentive period, she earned $0 for that sample and the incentive schedule was reset to the original voucher value of $7.50 · If the woman again met the target reduction on 5 consecutive occasions, she earned vouchers at the previously attained level  I_2_ = CAP-standard care plus non-contingent incentives:  · Financial incentives delivered by an already generated schedule, not linked to cigarette smoking | CAP-standard care:  · Information about the adverse effects associated with cigarette smoking  . Educational materials about risks of smoking during pregnancy  · During follow-up appointments, routinely asking about cigarette smoking and commended on efforts to abstain  · Compensation for urine and breath samples  · Brief motivational interviewing feedback session (10 min) | Biochemically verified smoking cessation (< 4 ppm CO in breath, < 200 ng/ml cotinine in urine) | **31 (I_1_) vs. 0 (I_2_) vs. 0%** |  | + |
|  |  |  |  |  |  | APGAR score 5 min | 8.7±1.7 (I_1_) vs. 8.8±0.6 (I_2_) vs. 8.6±1.4 |  | = |
|  |  |  |  |  |  | BW | 2863.3±694.3 (I_1_) vs. 2695.6±656.9 (I_2_) vs. 2701.3±598.3 g |  | = |
|  |  |  |  |  |  | GA at delivery | 37.9±3.6 (I_1_) vs. 37.0±3.0 (I_2_) vs. 37.5±3.2 weeks |  | = |
|  |  |  |  |  |  | LBW (< 2,500 g) | 20.0 (I_1_) vs. 37.5 (I_2_) vs. 42.9% |  | = |
|  |  |  |  |  |  | NICU admission | 46.7 (I_1_) vs. 50.0 (I_2_) vs. 61.9% |  | = |
|  |  |  |  |  |  | Preterm birth | 16.7 (I_1_) vs. 35.3 (I_2_) vs. 28.6% |  | = |
|  |  |  |  |  |  | Spontaneous abortion | 3 (I_1_) vs. 10 (I_2_) vs. 10% |  | = |
|  |  |  |  |  |  | Treated for neonatal abstinence syndrome | 87.1 (I_1_) vs. 66.7 (I_2_) vs. 81.0% |  | = |
| 5 | Glover et al. | 2015 | RCT | I_1_ = $25 voucher from Farmers Trading Company for each ‘abstinent from smoking’ week, for 8 weeks  I_2_ = Products to the value of $25 for each ‘abstinent from smoking’ week, for 8 weeks  · Women who did not remain abstinent from smoking were not provided with any more incentives  · $5 retention gift for each face-to-face visit paid upon completion of the final visit | Standard cessation support: information about different cessation products and services, and access to nicotine replacement therapy | Biochemically verified smoking abstinence (< 7 ppm CO in breath) | 21% (overall) |  | = |
| 5 | Heil et al. | 2008 | RCT | · $6.25 voucher upon submitting breath CO tests ≤ 6 ppm during the initial 5 days and urine-cotinine levels ≤ 80 ng/ml beginning in week 2 of abstinence monitoring  · $1.25 increase of voucher per consecutive negative tests (maximum: $45)  · Positive test results or missed visit set the voucher value back to the original low value, but 2 consecutive negative tests restored the value to the pre-reset level  · Redeemable for retail items | Standard cessation support plus non-contingent vouchers:  · $15 voucher per visit antepartum  · $20 voucher per visit postpartum | Biochemically verified smoking cessation at the end of pregnancy (< 6 ppm CO in breath, < 80 ng/ml cotinine in urine) | **41 vs. 10%** |  | + |
|  |  |  |  |  |  | BW | 3355±96 vs. 3102±89 g |  | = |
|  |  |  |  |  |  | GA at delivery | 39.1±0.4 vs. 38.5±0.3 weeks |  | = |
|  |  |  |  |  |  | LBW | 9 vs. 21% |  | = |
|  |  |  |  |  |  | NICU admissions | 12 vs. 15% |  | = |
|  |  |  |  |  |  | Preterm birth | 9 vs. 23% |  | = |
| ***Drug use*** | | | | | | | | | |
| 7 | Jones et al. | 2011 | RCT | CAP-standard care plus RBT:  · Individualized treatment planning, behavior graphing, weekly recreational, vocational, and peer reinforcement groups  · A woman’s only recovery house which allowed children to live in home on the assisted living unit day of discharge, and transportation  · If the women refused entering a recovery house: a drug-free living environment in order facilitate abstinence goals  · If drug/alcohol use was detected, either at the program or the house: an alternative living arrangement until the woman once again tested negative for opioids and cocaine  · Attendance CAP 7 days per week for the first month of outpatient treatment  · Individual counseling 2-3 times/week including behavior graph review and elements of vocational counseling  · Urine positive test resulted in an individual counseling session focused on relapse including a functional analysis, detailed day planning and problem-solving strategies  · Drug abstinent-contingent benefits were in effect for 6 months | CAP-standard care  · 7-night stay on an assisted living unit followed by daily intensive outpatient treatment  · Treatment for substance use disorders (group and individual counseling and psychoeducation), medically-assisted withdrawal for patients either refusing methadone maintenance or not meeting current opioid dependence criteria, methadone maintenance for qualifying opioid-dependent patients, case management, psychiatric evaluation and treatment, general medical management, on-site child care and pediatric care | Biochemically verified drug abstinence (< 300 ng/ml illicit opiates and cocaine in urine) | OR = 0.48 | 0.11 to 2.08 | = |
|  |  |  |  |  |  | BW | 2708.3±143.0 vs. 2996.3±198.8 g |  | = |
|  |  |  |  |  |  | GA at delivery | 37.2±1.1 vs. 38.5±1.6 weeks |  | = |
|  |  |  |  |  |  | Length of hospitalized stay after birth | **2.9±0.35 vs. 4.2±0.53 days** |  | + |
|  |  |  |  |  |  | Preterm birth (< 37 weeks of pregnancy) | OR = 0.85 | 0.21 to 3.39 | = |
| 5 | Jones et al. | 2001 | RCT | Escalating voucher schedule throughout the 7-day residential care phase and the first 7 days of the 30-day intensive outpatient phase:  · $5.00 voucher for emitting target behavior (residential care phase: ≥ 4 hours of individual/group counseling per day, outpatient care phase: ≥ 4 hours of individual/group counseling per day and cocaine negative urine samples)  · $5.00 increase of voucher per consecutive day that the target behavior(s) were met (maximum: $70 · Redeemable for merchandise and services purchased by research staff  · If women failed to attend one full treatment day, no voucher that day but the value of the next earned voucher would not be reset (all subsequent treatment absences and all cocaine positive urine samples would reset the incentive value to $5.00) | Standard care, comprehensive treatment program:  · 7-day residential care phase  · 30-day intensive outpatient phase (7 days per week, 6.5 hours per day)  · Group counseling with individual psychotherapy ≥ 1 times/week  · Child care services  · On-site obstetrical care, pediatric care, psychiatric consultation | Biochemically verified drug abstinence (< 300 ng/ml illicit opiates and cocaine in urine) |  |  | =  = |
| ***Dietary intake (and physical activity)*** | | | | | | | | | |
| 7 | Phillips et al. | 2019 | RCT | Individual sessions (each 30 min) every 2 weeks performed by a behaviorally trained nutritionist:  · Setting initial goal for daily calorie intake based on BMI and activity level  · Principles of behavioral weight management: self-monitoring, stimulus control, problem solving, motivation, feedback and social support  · Providing cash payments at each bi-monthly visit, contingent on the woman’s success of not exceeding GWG guidelines for GA (maximum: $550)  · Escalating schedule of reward for continued success, with a reset for failure to meet the goal | Standard care: advice to follow the IOM weight gain guidelines, to follow an exercise plan and nutrition consultation | GWG | 22.1±1.8 vs. 22.0±1.8 pounds |  | = |
|  |  |  |  |  |  | GWG in excess of the IOM weight gain guidelines | 30 vs. 29% |  | = |
|  |  |  |  |  |  | BW | 3,468±68 vs. 3,497±65g |  | = |
|  |  |  |  |  |  | GA at delivery | 39.2±0.2 vs. 39.5±0.2 weeks |  | = |
|  |  |  |  |  |  | GDM | OR = 0.65 | 0.21 to 1.99 | = |
|  |  |  |  |  |  | Hypertensive disorders of pregnancy | OR = 1.34 | 0.55 to 3.31 | = |
|  |  |  |  |  |  | LGA (BW > P90) | OR = 1.01 | 0.38 to 2.69 | = |
|  |  |  |  |  |  | Macrosomia (BW > 4,000 g) | OR = 1.97 | 0.80 to 4.84 | = |
|  |  |  |  |  |  | NICU admission | OR = 1.57 | 0.53 to 4.64 | = |
|  |  |  |  |  |  | Primary CS | OR = 0.58 | 0.24 to 1.43 | = |
|  |  |  |  |  |  | SGA (BW < P10) | OR = 0.59 | 0.10 to 3.68 | = |

Abbreviations: BW, birthweight; CAP, Center for Addiction and Pregnancy; CI, confidence interval; GA, gestational age; LBW, low birthweight; NICU, neonatal intensive care unit; OR, odds ratio; P10, 10^th^ percentile; P90, 90^th^ percentile; RBT; reinforcement based treatment; RR, relative risk; QS, quality score. *Relevant outcomes are presented as ratios, percentages, mean difference or as mean±SD.

Supplemental Table 1D. Description and summary of findings of studies investigating the effect of mindfulness.

| QS | Author | Year | Study design | Description of intervention(s) | Control situation | Relevant outcome* | | 95% CI | Main result |  |
| --- | --- | --- | --- | --- | --- | --- | --- | --- | --- | --- |
| *Dietary intake (and physical activity)* | | | | | | | | | | |
| 7 | Epel et al. | 2019 | Trial | 8 Mindful Moms Training sessions (each 120 min), 2 booster telephone sessions and 1 postpartum group session performed by trained practitioners:  · Check-in where each woman shared her experiences and mindful movement  · Didactic discussions covering stress reduction, mindful eating and nutrition  · Mindfulness practice and a review of homework for the upcoming week | Standard care: routine prenatal care | GWG | AD = 0.05 kg |  | = |  |
|  |  |  |  |  |  | GWG in excess of IOM weight gain guidelines | OR = 1.39 | 0.60 to 3.22 | = |  |

Abbreviations: AD, absolute difference; CI, confidence interval; GWG, gestational weight gain; IOM, Institute of Medicine; OR, odds ratio; QS, quality score. *Relevant outcomes are presented as ratios.

Supplemental Table 1E. Description and summary of findings of studies investigating the effect of hypnosis.

| **QS** | **Author** | **Year** | **Study design** | **Description of intervention(s)** | **Control situation** | **Relevant outcome*** | | **95% CI** | **Main result** |
| --- | --- | --- | --- | --- | --- | --- | --- | --- | --- |
| ***Smoking*** | | | | | | | | | |
| 7 | Valbo et al. | 1996 | RCT | 2 sessions (each 45 min) performed by a hypnotist (anesthesiologist):  · Conventional induction to a superficial non-somnambulistic stage of trance  · A tape to encourage the woman’s will and capacity to quit smoking  · Relaxation techniques and self-hypnotic methods to combat cravings | Standard care: routine prenatal care | Self-reported smoking abstinence | 10 vs. 10% |  | = |

Abbreviations: CI, confidence interval; RCT, randomized controlled trial; QS, quality score. *Relevant outcomes are presented as ratios, percentages, mean difference or as mean±SD.
